# Predicting the Redox Potentials and Hammett Parameters of Quinone Derivatives with the Information-Theoretic Approach

**DOI:** 10.3390/e28010067

**Published:** 2026-01-06

**Authors:** Mingxin Xu, Yilin Zhao, Hui Li, Paul W. Ayers, Dandan Liu, Qingchun Wang, Dongbo Zhao

**Affiliations:** 1 School of Pharmaceutical Sciences and Yunnan Key Laboratory of Pharmacology for Natural Products, Kunming Medical University, Kunming 650500, China; 201913079@kmmu.edu.cn; 2 Department of Chemistry and Chemical Biology, McMaster University, Hamilton, ON L8S 4M1, Canada; zhaoyilin10@foxmail.com (Y.Z.); ayers@mcmaster.ca (P.W.A.); 3 School of Life Sciences, Yunnan University, Kunming 650500, China; lihuilyj@163.com; 4 Institute of Artificial Intelligence, Hefei Comprehensive National Science Center, Hefei 230088, China; 5 Institute of Biomedical Research, Yunnan University, Kunming 650500, China

**Keywords:** quantum machine learning (QML), deep learning (DL), information-theoretic approach (ITA), redox potential, Hammett parameter

## Abstract

Accurately and efficiently predicting redox potentials and Hammett constants using simple density-based functions derived from information-theoretic approach (ITA) quantities remains an unresolved challenge. In this work, we employ two recently proposed protocols, DL(ITA) (deep learning) and QML(ITA) (quantum machine learning), to a broad range of quinone derivatives with available experimental data. The molecular electrostatic potential (MEP) at the nucleus of the acidic atom and the sum of valence natural atomic orbital (NAO) energies are used within a linear regression (LR) framework to assess the first redox potentials and Hammett parameters of these quinone derivatives. The DL(ITA) protocol enables the construction of a transferable model trained on quinone derivatives that can be applied to both quinone and non-quinone systems. Interestingly, the QML(ITA) model exhibits superior performance compared to the DL(ITA) approach. Moreover, the structure of the QML(ITA) method suggests that it may be readily implemented on real quantum hardware in the near future.

## 1. Introduction

Redox-active molecules, such as quinones and phenazines, play essential roles as cathodic and anodic species in organic battery systems [1,2]. The redox potential is a fundamental physicochemical quantity that dictates the direction and efficiency of electron transfer processes [3]. Experimentally, Refs. [4,5,6,7] show that redox potentials are usually obtained by cyclic voltammetry when reversible voltammetric behavior is observed. Theoretically, Refs. [8,9,10,11,12,13,14,15,16] show that they are often computed using ab initio quantum chemical methods based on thermodynamic cycles. However, these approaches require detailed modeling of experimental environments, resulting in non-negligible uncertainties and computational overhead.

To address these limitations, several studies [17,18,19] have explored alternative strategies based on simple electronic descriptors. Molecular electrostatic potentials (MEPs) and natural atomic orbital (NAO) energies have been shown to enable efficient and reasonably accurate predictions of acidity constants (p*K*_a_) and Hammett parameters (*σ*_p_) of benzoic acids using only gas-phase information. Nevertheless, these efforts do not yet constitute a broadly applicable theoretical framework. This motivates a central question: Can simple density-based descriptors be systematically combined to construct a unified model for predicting experimental first and second redox potentials as well as Hammett parameters? In this work, we provide an affirmative answer.

**Scheme 1:**
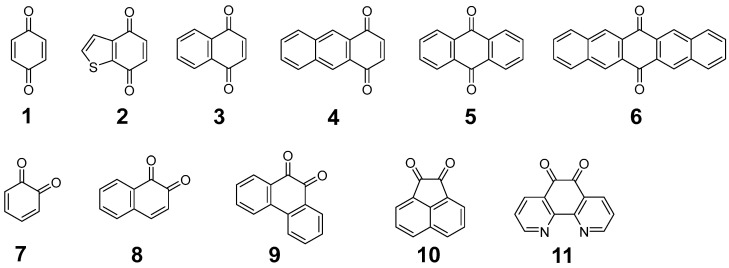
*Para*-quinones (**1**–**6**) and *ortho*-quinones (**7**–**11**) form the basis of this work. **1**: 1,4-benzoquinone, **2**: 4,7-benzo[*b*]thiophenequinone, **3**: 1,4-naphthoquinone, **4**: 1,4-anthraquinone, **5**: 9,10-anthraquinone, **6**: 6,13-pentacenequinone, **7**: 1,2-benzoquinone, **8**: 1,2-naphthoquinone, **9**: 9,10-phenanthrenequinone, **10**: 1,2-acenapthenequinone, and **11**: 1,10-phenanthroline-5,6-quinone.

**Scheme 2:**
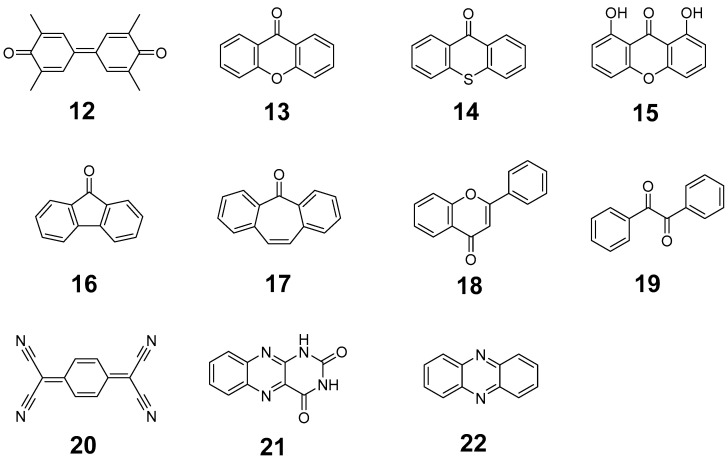
Non-quinone (**12**–**19**) and nitrogen-containing (**20**–**22**) species. **12**: 3,3′,5,5′-tetramethyl-4,4′-diphenoquinone, **13**: 1,4-dihydroxy-xanthen-9-one, **14**: thiaxanthene-9-one, **15**: 1,8-dihydroxy-9-anthrone, **16**: fluorenone, **17**: dibenzo[*a*,*d*]cyclohepten-5-one, **18**: flavone, **19**: benzil, **20**: 7,7,8,8-tetracyano–1,4-quinonedimethane, **21**: alloxazine, and **22**: phenazine.

**Figure 1:**
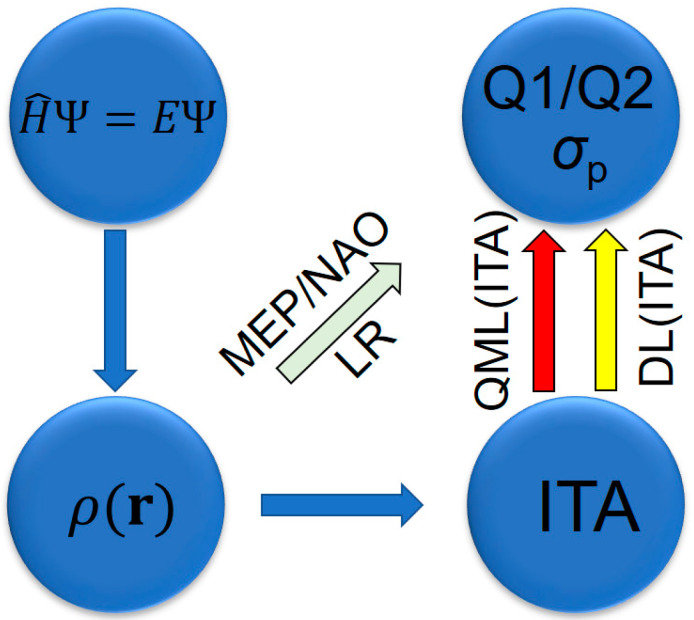
Workflow of the QML(ITA), DL(ITA), and LR (linear regression) methods in predicting the 1st and 2nd experimental redox potentials (Q1/Q2) and Hammett constants (*σ*_p_). Molecular electrostatic potential (MEP) and natural atomic orbital (NAO) energies are used as input for LR. Density-based ITA quantities and HOMO/LUMO energies are used as input for QML(ITA) and DL(ITA).

Building on our previous studies that employed density-based quantities to interpret various physicochemical properties, including molecular polarizabilities [20,21,22,23,24,25,26], NMR shielding constants [25], excitation energies [24,26], log*K*_ow_ values [26], and *post*-Hartree–Fock correlation energies [26,27,28], we extend this line of research to a diverse set of quinone and non-quinone systems (Scheme 1 and Scheme 2). We introduce a collection of information-theoretic descriptors derived from the information-theoretic approach (ITA) [29,30] within density functional theory (DFT) [31,32], including Shannon entropy [33], Fisher information [34], relative Shannon entropy [35], relative Fisher information [36], Onicescu information energy [37], relative Rényi entropy [37], and GBP entropy [38]. These physics-inspired density-based descriptors form the feature space for each molecular system and are subsequently processed using classical deep learning (DL) or quantum machine learning (QML), as summarized in Figure 1. Our results showcase that linear regression (LR) yields system-dependent predictions, whereas both DL(ITA) [25,26] and QML(ITA) [39,40] achieve system-independent performance. This highlights the generalization capability of ITA descriptors when combined with modern machine learning (ML) methods and underscores their potential for broader applications in molecular property prediction.

## 2. Results

Shown in Tables S1 and S2 are the molecular electrostatic potential on two oxygen atom nuclei (MEP@O), sum of valence NAO energies of two oxygen atoms (ΣNAO@O), and the experimental first/second redox potentials (Q1/Q2) [41] and Hammett parameters (*σ*_p_) [42], for a total of 116 1,4-benzoquinone derivatives, which are calculated at the B3LYP/def2-TZVP [43,44,45] level of theory. For the other molecules, the relevant data are provided in Tables S3–S5.

**Scheme 3:**
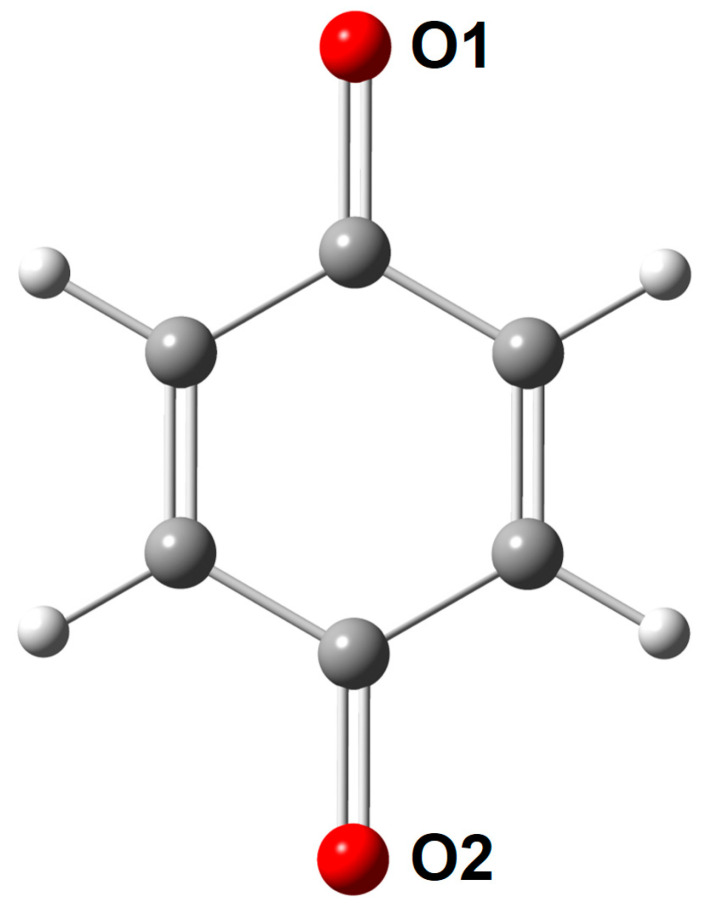
The quinone scaffold showing the two carbonyl oxygen atoms, denoted as O1 and O2, where the molecular electrostatic potential at the nuclear position is computed. Color code: H in white, C in gray, and O in red.

**Figure 2:**
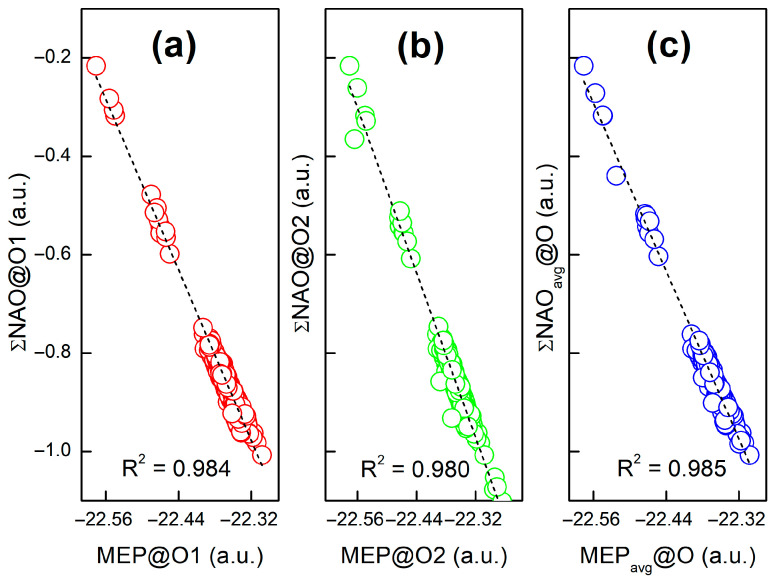
Strong correlations between the molecular electrostatic potential at the nuclei of two oxygen atoms (**a**,**b**) attached to the C=O group and (**c**) their average and the sum of natural atomic orbital energies of the same oxygen atom for all the species considered in this work. The *y*-axis spans from (**a**–**c**).

**Figure 3:**
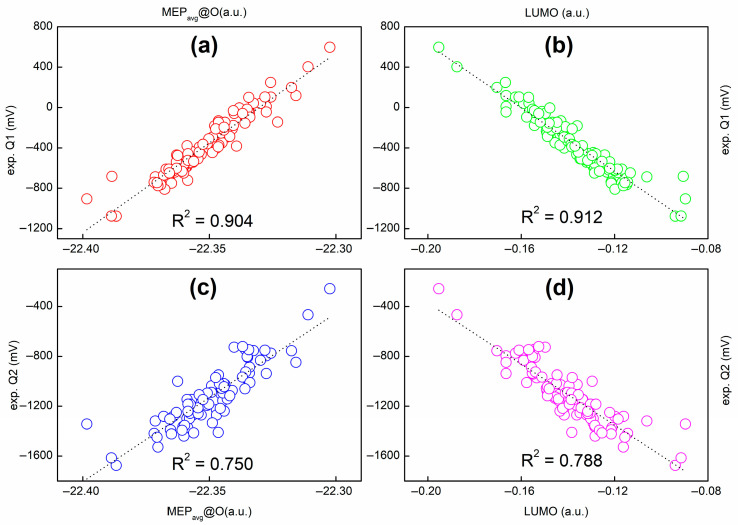
Linear relationships between (**a**,**c**) molecular electrostatic potential at the nucleus of oxygen, (**b**,**d**) lowest unoccupied molecular orbital (LUMO) energies, and the experimental 1st (Q1) and 2nd (Q2) redox potentials, for a total of 1165 1,4-benzoquinone (BQ) derivatives.

Shown in Figure 2 are strong correlations between molecular electrostatic potential at the nuclei of two oxygen atoms [(a) and (b)] belonging to the carbonyl groups of the quinone scaffold, denoted as O1 and O2 (see Scheme 3), and (c) their average and the sum of natural atomic orbital energies of the same oxygen atom for all the species considered in this work. This observation is in line with our work on molecular acidity [17,18,19]. In Figure 3, we plot the MEP@O_avg_ and lowest unoccupied molecular orbital (LUMO) energies for the experimental first (Q1) and second (Q2) redox potentials, for a total of 116 1,4-benzoquinone (BQ) derivatives. The corresponding correlation coefficients (*R*^2^) are 0.904 and 0.912 for Q1, and 0.750 and 0.788 for Q2. Of note, a single strong correlation is not observed for all the species, indicating that MEP or LUMO alone cannot be a universal descriptor of Q1/Q2. Plus, the linear regression (LR) model cannot be used to predict the Q1/Q2 data. This inspires us to construct a non-linear model, as we will show below.

**Figure 4:**
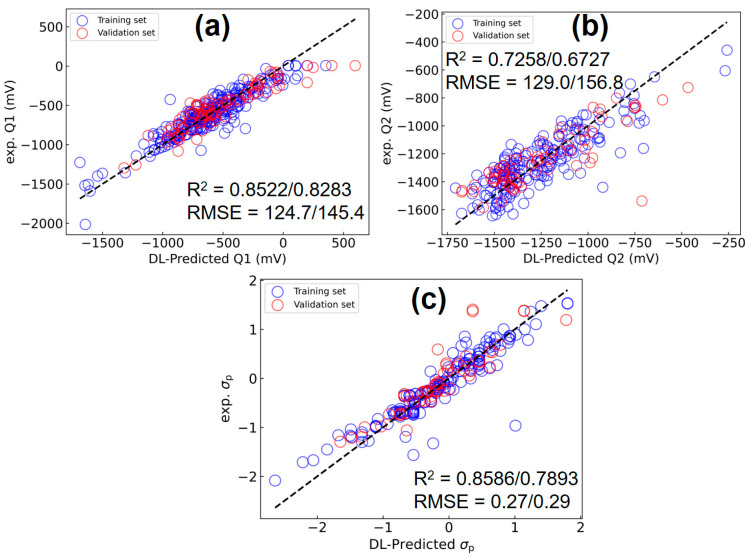
Comparison of the DL-predicted and experimental (**a**) Q1, (**b**) Q2, and (**c**) *σ*_p_. A total of 11 ITA quantities and HOMO/LUMO energies are used as inputs for DL(ITA). RMSE: root mean squared error. *R*^2^: correlation coefficient.

With 11 ITA quantities (see Table S2) and HOMO/LUMO energies as inputs, the DL(ITA) model can simultaneously predict the experimental Q1 and Q2 data, as well as Hammett constants *σ*_p_, as shown in Figure 4. For the other molecules, the ITA data are provided in Tables S6–S8. Plus, strong linear correlations between ITA quantities, (a) $R_{3}^{r}$ vs. $R_{2}^{r}$, (b) G_2_ vs. G_1_, and (c) I_G_ vs. G_3_, for all the species considered are shown in Figure S1. For Q1/Q2, the root mean squared errors (RMSEs) are 124.7/145.4 and 129.0/156.87 mV for the training and validation sets, respectively. Of note, while the mean absolute errors (MAEs) for Q1 reported in the literature range from 83.29 to 89.51 mV [16], our results are in the range of 94.97~99.89 mV. Notably, our multi-property DL(ITA) model achieves comparable accuracy using only a single gas-phase calculation of the neutral species, highlighting its computational efficiency over conventional methods. For *σ*_p_, the RMSEs are 0.27 and 0.29, respectively. These data collectively demonstrate that the DL(ITA) model outperforms its LR counterpart.

**Figure 5:**
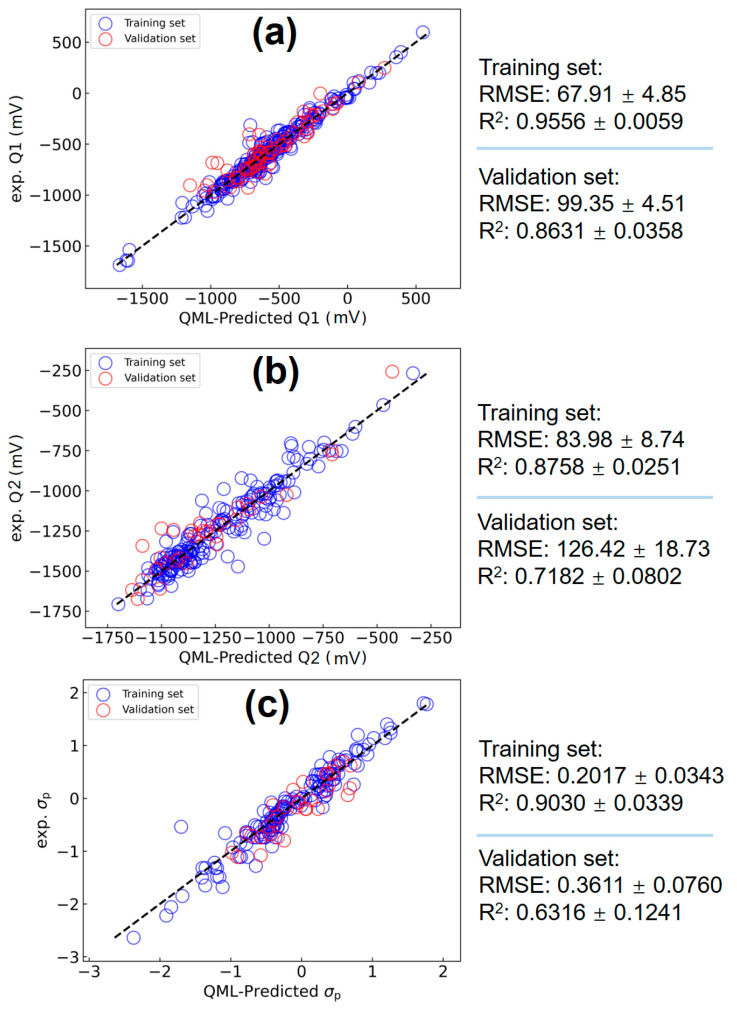
Comparison of the QML-predicted and experimental (**a**) Q1, (**b**) Q2, and (**c**) *σ*_p_ data. A total of 11 ITA quantities and HOMO/LUMO energies are used as inputs for QML(ITA). Five-fold cross-validation is used, and the reported results correspond to the best-performing fold among the five-fold cross-validation, rather than the averaged results across all folds. The error bars are not intended to represent statistical uncertainty (e.g., standard deviation across folds). RMSE: root mean squared error. *R*^2^: correlation coefficient.

Next, we switch our gear to the QML(ITA) model, which is applied for the first time to predict the Q1/Q2 and *σ*_p_ data. Figure 5 compares the QML(ITA)-predicted and experimental values for Q1 (Figure 5a), Q2 (Figure 5b), and *σ*_p_ (Figure 5c). The accuracy for Q2 and *σ*_p_ is similar to that obtained from DL(ITA), but the Q1 predictions are further improved: the root mean squared errors (RMSEs) for Q1 are 67.91 and 99.35 mV for the training and validation sets, respectively. These results indicate that the QML(ITA) model provides superior accuracy for Q1 while retaining the advantages of a single, neutral gas-phase calculation.

## 3. Discussion

In this work, we introduce two approaches, QML(ITA) and DL(ITA), for predicting the first and second experimental redox potentials as well as the Hammett constants of a broad range of quinone derivatives. While the DL(ITA) model has previously been applied to the first redox potential of phenazine derivatives [26], the present study constitutes the first introduction of the QML(ITA) method within a hybrid quantum–classical framework [39,40]. In the current implementation, both the construction of ITA data and the execution of QML algorithms are performed on classical computers. Unlike conventional system-dependent LR models, our results show that QML(ITA) and DL(ITA) are system-independent. Models trained exclusively on quinones can successfully predict properties of non-quinones, highlighting the generalizability of the ITA-based methods. Nevertheless, the models are not yet universal due to the limited amount of experimental data available for training.

While our results show that the QML model combined with the information-theoretic approach [QML(ITA)] slightly outperforms the deep learning variant [DL(ITA)] for predicting Q1, the improvement in absolute accuracy is modest. This raises the question of whether the additional complexity of a quantum-inspired model justifies its use. From a computational standpoint, QML(ITA) generally requires more resources due to the preparation and manipulation of variational quantum circuits, whereas DL(ITA) benefits from mature, optimized classical frameworks that scale efficiently to larger datasets. On the other hand, QML models often provide better interpretability, since the circuit structure can reflect underlying physical or chemical relationships, whereas DL models, especially with large networks, tend to be black boxes. Scalability is another consideration. While QML may offer advantages for systems with a strong quantum character or where data is limited, DL approaches are easier to scale across diverse chemical spaces. Overall, the choice between QML(ITA) and DL(ITA) should weigh these trade-offs; modest predictive gains may be offset by higher computational cost, but the potential for mechanistic insight and principled model design could justify QML in certain contexts.

A central question is why conceptually simple model architectures are capable of simultaneously predicting multiple physicochemical properties, including the first and second redox potentials and Hammett constants. The answer lies in the ability of ITA descriptors to encode essential information about the electron density, particularly its degree of (de)localization. According to the first Kohn–Sham theorem [46,47], the electron density uniquely determines all ground-state molecular properties. Consequently, mapping the electron density to ITA quantities provides a compact and distinct molecular representation. This work, therefore, goes beyond a direct extension of our earlier DL(ITA) model; instead, we aim to construct a general ITA-based ML framework capable of predicting a wide range of physicochemical properties. Ultimately, we will implement these models on actual quantum computers in the near future.

In addition, one of the coauthors has outlined four fundamental criteria required for ML features to be capable of simulating arbitrary molecular properties [48]. The most stringent one states that the ML features must be able to reproduce the ground-state electron density, as density-functional theory guarantees that all ground-state (and even low-lying excited-state) properties are functionals of the electron density. If the ML features can reproduce the electron density, then ML models based on them should, in principle, be able to predict any property of the system. Several strategies could, in principle, satisfy this criterion, such as using density moments [49] or other descriptors. Recently, using ITA descriptors, we successfully predicted frontier orbital energies and various energetic and structural properties [50]. Building on these results, we proposed “information functional theory” [51], stating that ITA quantities form a complete set capable of reconstructing the electron density and thus predicting any physicochemical property. We further introduce a novel concept of an “ITA–ML Loop”, where low-tier ITA quantities are used to train ML models that yield high-quality electron densities, which are then used to compute high-tier (e.g., DFT-level) ITA quantities.

A recent study [52] also points out that quantum mechanics-based descriptors are particularly advantageous when (*i*) the training set contains < 2000 data points, (*ii*) the descriptors correlate strongly with the target properties, and (*iii*) the descriptors can be computed with reasonable accuracy and cost. ITA descriptors satisfy criteria (*ii*) and (*iii*), and most experimental datasets naturally satisfy (*i*). Our findings suggest that ITA-based ML techniques should be applied to more challenging systems, such as porphyrin nanorings, condensed-phase systems with significant solvent effects, and strongly correlated systems [27]. For condensed-phase systems, such as liquid water or molecular crystals, *local* ITA descriptors may be needed, potentially together with convolutional neural networks, while in this work, *global* ITA quantities already exhibit good performance for molecular systems. Furthermore, incorporating graph-theoretic information [53] may enrich the ITA-DL framework and enable qualitative comparisons with other soft-computing methodologies [54].

## 4. Materials and Methods

### 4.1. Information-Theoretic Approach (ITA) Quantities

Shannon entropy *S*_S_ [33] and Fisher information *I*_F_ [34] are two cornerstone quantities in information theory. They are defined as Equations (1) and (2), respectively.

(1) $$S_{S}=-\int \rho \left(r\right)ln\rho \left(r\right)dr$$

(2) $$I_{F}=\int \frac{{\left|\nabla \rho (r)\right|}^{2}}{\rho (r)}dr$$

where $\rho \left(r\right)$ is the electron density and ∇ρ(r) is the density gradient. The physical picture of $S_{S}\;$ and $I_{F}$ is clear; the former measures the spatial delocalization of the electron density, and the latter gauges the sharpness or localization of the same.

Except for the total density, more ingredients, such as kinetic energy density, can be used to define an ITA quantity. With both the electron density and the kinetic energy density, Ghosh, Berkowitz, and Parr developed a formula for entropy (S_GBP_) [38] as follows:

(3) $$S_{GBP}=-\int \frac{3}{2}k\rho \left(r\right)\left[c+ln\frac{t(r;\rho )}{t_{TF}(r;\rho )}\right]dr$$

where *t*(**r**; *ρ*) and *t*_TF_(**r**; *ρ*) represent the non-interacting and Thomas–Fermi (TF) kinetic energy density, respectively. Here *k*, *c*, and *c*_K_ are three constants [*k*, the Boltzmann constant, *c* = (5/3) + ln(4π*c*_K_/3), and *c*_K_ = (3/10)(3π^2^)^2/3^]. Full integration of the kinetic energy density *t*(**r**; *ρ*) leads to the total kinetic energy *T*_S_ via

(4) $$\int t\left(r;\rho \right)dr=T_{S}$$

while *t*(**r**; *ρ*) can be obtained from the canonical orbital densities as follows:

(5) $$t\left(r;\rho \right)=\sum_{i}\frac{1}{8}\frac{\nabla \rho_{i}\cdot \nabla \rho_{i}}{\rho_{i}}-\frac{1}{8}\nabla^{2}\rho$$

and *t*_TF_(**r**; *ρ*) is simply cast in terms of $\rho \left(r\right)$ as follows:

(6) $$t_{TF}(r;\rho )=c_{K}\rho^{5/3}\left(r\right)$$

Of note, the kinetic energy density can differ in its form and thus can be used in different contexts [55,56,57,58,59,60,61,62]. But, S_GBP_ satisfies the maximum-entropy requirement from a mathematical viewpoint [38].

Moving forward, some ITA quantities have been introduced for chemical reactions. As new reactivity descriptors in conceptual density functional theory (CDFT) [63,64,65,66], one example is relative Rényi entropy [37] (of order *n*) as follows:

(7) $$R_{n}^{r}=\frac{1}{1-n}{log}_{10}\left[\int \frac{\rho^{n}\left(r\right)}{\rho_{0}^{n-1}\left(r\right)}dr\right]$$

Information gain [35] (also called Kullback−Leibler divergence or relative Shannon entropy) *I*_G_ is expressed as follows:

(8) $$I_{G}=\int \rho \left(r\right)ln\frac{\rho \left(r\right)}{\rho_{0}\left(r\right)}dr$$

In Equations (7) and (8), $\rho_{0}\left(r\right)$ and *ρ*(**r**) satisfy the same normalization condition, and $\rho_{0}\left(r\right)$ denotes the reference-state density.

Recently [36], one of the present authors proposed three functions, *G*_1_, *G*_2_, and *G*_3_, at both atomic and molecular levels. They are defined as follows:

(9) $$G_{3}=\sum_{A}\int \rho_{A}\left(r\right){\left[\nabla ln\frac{\rho_{A}\left(r\right)}{\rho_{A}^{0}\left(r\right)}\right]}^{2}dr$$

(10) $$G_{1}=\sum_{A}\int {\nabla^{2}\rho }_{A}\left(r\right)\frac{\rho_{A}\left(r\right)}{\rho_{A}^{0}\left(r\right)}dr$$

(11) $$G_{2}=\sum_{A}\int \rho_{A}\left(r\right)\left[\frac{{\nabla^{2}\rho }_{A}\left(r\right)}{\rho_{A}\left(r\right)}-\frac{\nabla^{2}\rho_{A}^{0}\left(r\right)}{\rho_{A}^{0}\left(r\right)}\right]dr$$

Equations (9)–(11) have been theoretically derived, numerically verified, and have witnessed many applications. Note that *G*_1_ and *G*_3_ are closely related to the density deformation, which can be utilized to quantify various chemical processes and transformations. *G*_2_ involves Laplacian contribution and can be regarded as the relative Laplacian contribution to the steric potential [67,68,69]. One of our major achievements during the past decade is when we aimed to glue the density functional theory and information theory together in a seamless manner because these two theories both can have electron density as inputs. Our recent progress along this line can be found in three reviews [70,71,72]. As another prominent example, we have applied ITA to appreciate homochirality [73,74].

Finally, Hirshfeld’s stockholder approach [75,76] is often introduced to partition atoms in a molecule in the literature, as defined in Equation (12) as follows:

(12) $$\rho_{A}\left(r\right)=\omega_{A}\left(r\right)\rho \left(r\right)=\frac{\rho_{A}^{0}\left(r\right)(r-R_{A})}{\sum_{B}\rho_{B}^{0}\left(r-R_{A}\right)}\rho \left(r\right)$$

Here, ${\;\rho }_{A}\left(r\right)$ is the atomic Hirshfeld density, $\omega_{A}\left(r\right)$ is a sharing function, and $\rho_{B}^{0}\left(r-R_{A}\right)$ is the atomic density of B centered at **R**_A_. The sum of all the free atom densities, typically spherically averaged S_0_ atomic densities, is normally termed the promolecular density. The Stockholder approach is natural in the context of ITA because it is also based on information-theoretic arguments. Alternative partitioning schemes include Becke’s fuzzy atom approach [77] and Bader’s zero-flux atoms-in-molecules (AIM) method [78].

### 4.2. Computational Details

Experimental Q/Q•^−^ (Q1) and Q•^−^/Q^2−^ (Q2) reduction potentials were reported recently by Prince et al. in dry *N*,*N*-dimethylformamide solvent against a saturated calomel electrode [41]. Most molecules are derivatives of 1,4-benzoquinone (BQ), 1,4-naphthoquinone (NQ), and 9,10-anthraquinone (AQ), as well as some aromatic non-quinones. Specifically, Prince et al. reported 350 experimental reduction potentials for 117 substituted BQs, 90 substituted NQs, 110 substituted AQs, and 33 miscellaneous quinones and non-quinones. They are labeled using indices 000 to 349. Of note, 3 (out of 350) molecules are excluded: 3,x-dichloro-2-methoxycarbonyl–1,4-benzoquinone (116), where a structure could not be determined from its name, and doxorubicin (209) and cerubidine (210), which are the same as adriamycin (192) and cerubidine (193). This resulted in a total of 347 Q1 reduction potentials. Experimental Q2 reduction potentials were not reported for 80 out of the 347 molecules, so we have 265 Q2 reduction potentials. Experimental Hammett parameters were taken from Ref. [42].

All density functional theory (DFT) computations were carried out with the Gaussian 16 (Revision C01) [79] package. First, all the molecular structures were optimized in the gas phase at the B3LYP/def2-SVP [43,44,45] level without any symmetry constraints. Suffice to note that for the def2 basis sets, the pseudopotentials are inherently included for Br and I. The optimized Cartesian coordinates of all the systems are provided in the Supplementary Materials. For a molecular system with multiple local minima, the one with the lowest energy is chosen for further computations. Molecular wavefunctions (thus electron density) were obtained at the B3LYP/def2-TZVP [43,44,45] level in the Gaussian 16 formatted checkpoint format. For each molecule, a total of 11 ITA quantities were subsequently numerically integrated with the Multiwfn 3.8 [80,81] program. The reference atomic densities were obtained at the restricted open-shell B3LYP/def2-TZVP.

### 4.3. Classical Deep Learning (DL)

For each molecule, a total of 11 ITA quantities and the highest occupied molecular orbital (HOMO) energy and lowest unoccupied molecular orbital (LUMO) energy are used as feature space. The classical deep learning (DL) process is performed with the PyTorch (version 2.7.1) [82] package. The model is constructed with a sequential neural network with one input layer, a hidden layer, and an output layer. The rectified linear unit (ReLU) function is applied as an activation function, and each hidden layer consists of 6 neurons. The mean square error (MSE) quantity was used as the loss function, and the adaptive moment estimation (Adam) [83] algorithm was used with the ReduceLROnPlatea scheduler to optimize the model. To learn the details of code implementation, please check our domestic package named PyITA, which can be found on a public ITchem (version 0.1.0) repository: https://github.com/ZhaoYilin/itchem (accessed on 6 September 2025).

### 4.4. Quantum Machine Learning (QML)

In this study, we developed a hybrid quantum-classical model based on a variational quantum circuit (VQC). The preprocessing phase included standardization of both input features and target variables using established methods in scikit-learn (version 1.7.0) [84]. The dataset was partitioned into training and validation sets via cross-validation, with data loaders constructed using PyTorch’s dataset processing tool to facilitate batch processing and shuffling during training. The quantum model featured a VQC using PyVQNet (via PennyLane version 0.41.1) [85,86], integrated into a PyTorch-based hybrid architecture. This design takes full advantage of quantum computing’s potential advantages in feature mapping and entangled representation to enhance the model’s ability to express complex features. The classical component consisted of two fully connected layers that processed the 13 input features into a 12-dimensional representation suitable for encoding into the quantum circuit. This design not only helps extract high-level features but also ensures that the output dimension matches the encoding capabilities of the quantum circuit, facilitating the execution of efficient feature encoding. The quantum component utilizes 12 qubits, adopting an Angle Embedding strategy to encode classical features into quantum states. This encoding approach takes full advantage of the high-dimensional characteristics of Hilbert space to improve data separability [39,40].

Subsequently, two layers of basic entangling structures with *RX* rotation gates were introduced to establish correlations between qubits and generate entangled states, thereby enhancing the representational power of the system. The *RX* gates in each layer introduce trainable rotation operations to adjust the position of the quantum state on the Bloch sphere. These parameterized rotations are subsequently followed by controlled gate operations that establish quantum correlations between qubits, forming a structured entanglement pattern that enhances the model’s representational capacity. The circuit thus reproduces the non-linear interactions typical of classical neural networks. Following this, expectation values of the Pauli-*Z* operator were measured for all qubits, capturing the statistical properties of the quantum state to form a set of effective quantum features. These features were finally fed into a linear output layer to complete regression or classification tasks. The entire hybrid model was trained for 200 epochs using the same optimization strategy and loss function as the baseline classical model, ensuring comparability and interpretability of the results.

## 5. Conclusions

To summarize, we have applied the QML(ITA) and DL(ITA) methods to accurately and efficiently predict the experimental first and second redox potentials, as well as the Hammett parameters, of a wide range of quinone and non-quinone derivatives. In contrast to the conventional system-dependent linear regression (LR) approach, both QML(ITA) and DL(ITA) exhibit system-independent performance, owing to their density-based representations. Moreover, the newly proposed QML(ITA) method demonstrates somewhat better predictive accuracy than our previously developed DL(ITA) model. Finally, we note that the QML(ITA) algorithm will be implemented on real quantum chips in future work, and the corresponding results will be reported elsewhere.

## Data Availability

Data are contained within the article.

## Supplementary Materials

The following supporting information can be downloaded at https://www.mdpi.com/article/10.3390/e28010067/s1, Figure S1: Intercorrelations between ITA quantities: (a) $R_{3}^{r}$ vs. $R_{2}^{r}$, (b) G_2_ vs. G_1_, and (c) I_G_ vs. G_3_ for all the species considered in this work; Table S1: Molecular electrostatic potential on two oxygen atom nuclei (MEP@O), sum of valence NAO energies of two oxygen atoms (ΣNAO@O), and the experimental 1st/2nd redox potential (Q1/Q2, in mV) and Hammett parameters (*σ*_p_), for a total of 116 1,4-benzoquinone derivatives, calculated at the B3LYP/def2-TZVP level of theory; Table S2: Eleven information-theoretic (ITA) descriptors for 1,4-benzoquinones, calculated at the B3LYP/def2-TZVP level. All values are reported in atomic units; Table S3: Molecular electrostatic potential on two oxygen atom nuclei (MEP@O), sum of valence NAO energies of two oxygen atoms (ΣNAO@O), and the experimental 1st/2nd redox potential (Q_1_/Q_2_, in mV) and Hammett parameters (*σ*_p_), for a total of (108 out of 110) 9,10-anthraquinone derivatives, calculated at the B3LYP/def2-TZVP level of theory; Table S4: Molecular electrostatic potential on two oxygen atom nuclei (MEP@O), sum of valence NAO energies of two oxygen atoms (ΣNAO@O), and the experimental 1st/2nd redox potential (Q_1_/Q_2_, in mV) and Hammett parameters (*σ*_p_), for a total of 90 1,4-naphthoquinone derivatives, calculated at the B3LYP/def2-TZVP level of theory; Table S5: Molecular electrostatic potential on two oxygen atom nuclei (MEP@O), sum of valence NAO energies of two oxygen atoms (ΣNAO@O), and the experimental 1st/2nd redox potential (Q_1_/Q_2_, in mV), for a total of 33 miscellaneous quinone and nonquinone derivatives, calculated at the B3LYP/def2-TZVP level of theory; Table S6: Eleven information-theoretic (ITA) descriptors for 9,10-anthraquinone derivatives, calculated at the B3LYP/def2-TZVP level. All values are reported in atomic units; Table S7: Eleven information-theoretic (ITA) descriptors for 1,4-naphthoquinone derivatives, calculated at the B3LYP/def2-TZVP level. All values are reported in atomic units; Table S8: Eleven information-theoretic (ITA) descriptors for miscellaneous quinone and nonquinone derivatives, calculated at the B3LYP/def2-TZVP level. All values are reported in atomic units.
