# Supplementary material for: Predicting the Redox Potentials and Hammett Parameters of Quinone Derivatives with the Information-Theoretic Approach
Source: Entropy (Basel). 2026 Jan 6;28(1):67. doi: 10.3390/e28010067 (PMC12839887; doi:10.3390/e28010067)
Supplement: Supplementary file 1 [file entropy-28-00067-s001.zip › entropy-4071476-supplementary.pdf]

Supplementary information for

Predicting the Redox Potentials and Hammett Parameters of Quinone Derivatives with the Information-Theoretic Approach

Mingxin Xu [1,†], Yilin Zhao [2,†], Hui Li [3,†], Paul W. Ayers [2], Dandan Liu [1,*], Qingchun Wang [4,*] and Dongbo Zhao [5,*]

[1] School of Pharmaceutical Sciences and Yunnan Key Laboratory of Pharmacology for Natural Products,
Kunming Medical University, Kunming 650500, China;

[2] Department of Chemistry and Chemical Biology, McMaster University, Hamilton, ONL8S4M1, Canada;

[3] School of Life Sciences, Yunnan University, Kunming 650500, China;

[4] Institute of Artificial Intelligence, Hefei Comprehensive National Science Center, Hefei 230088, China;

[5] Institute of Biomedical Research, Yunnan University, Kunming 650500, China.

*Correspondence: liudandan@kmmu.edu.cn (D.L.); qingchun720@ustc.edu.cn (Q.W.); dongbo@ynu.edu.cn (D.Z.)

†These authors contributed equally to this work.

Table S1. Molecular electrostatic potential on two oxygen atom nuclei (MEP@O), sum of valence NAO energies of two oxygen atoms (ΣNAO@O), and the experimental 1st/2nd redox potential (Q1/Q2, in mV) and Hammett parameters (σ_P), for a total of 116 1,4-benzoquinone derivatives, calculated at the B3LYP/def2-TZVP level of theory.

entry	MEP@O$_1$	MEP@O$_2$	MEP@O$_{avg}$	ΣNAO@O$_1$	ΣNAO@O$_2$	ΣNAO@O$_{avg}$	exp. Q1	exp. Q2	exp. σ_P
000	–22.3268	–22.3282	–22.3275	–0.9392	–0.9353	–0.9373	10	–796	0.66
001	–22.3110	–22.3110	–22.3110	–0.9827	–0.9827	–0.9827	403	–466	1.32
002	–22.3024	–22.3024	–22.3024	–1.0073	–1.0073	–1.0073	597	–258	1.78
003	–22.3380	–22.3439	–22.3410	–0.9095	–0.8931	–0.9013	–212	–1073	0.23
004	–22.3471	–22.3473	–22.3472	–0.8856	–0.8977	–0.8916	–309	–1229	0.03
005	–22.3406	–22.3502	–22.3454	–0.9089	–0.8758	–0.8924	–284	–1104	0.06
006	–22.3424	–22.3519	–22.3472	–0.9096	–0.8711	–0.8904	–402	–1270	0.03
007	–22.3495	–22.3524	–22.3509	–0.8910	–0.8765	–0.8838	–398	–1193	–0.14
008	–22.3268	–22.3282	–22.3275	–0.9392	–0.9353	–0.9373	–44	–938	0.46
009	–22.3357	–22.3357	–22.3357	–0.9162	–0.9162	–0.9162	–108	–972	0.46
010	–22.3347	–22.3347	–22.3347	–0.9192	–0.9192	–0.9192	–84	–942	0.46
011	–22.3283	–22.3400	–22.3342	–0.9366	–0.9038	–0.9202	–53	–935	0.46
012	–22.3289	–22.3393	–22.3341	–0.9384	–0.9058	–0.9221	–45	–887	0.46
013	–22.3310	–22.3403	–22.3357	–0.9378	–0.9030	–0.9204	–75	–925	0.36
014	–22.3405	–22.3449	–22.3427	–0.9181	–0.8903	–0.9042	–195	–1136	0.26
015	–22.3410	–22.3454	–22.3432	–0.9193	–0.8912	–0.9053	–208	–1127	0.26
016	–22.3425	–22.3469	–22.3447	–0.9188	–0.8906	–0.9047	–234	–1156	0.16
017	–22.3432	–22.3426	–22.3429	–0.8954	–0.9095	–0.9025	–292	–1142	0.12
018	–22.3488	–22.3488	–22.3488	–0.9000	–0.9000	–0.9000	–447	–1334	0.06
019	–22.3436	–22.3447	–22.3442	–0.9040	–0.9059	–0.9050	–267	–1239	0.14
020	–22.3457	–22.3457	–22.3457	–0.9041	–0.9041	–0.9041	–254	–1217	0.16
021	–22.3464	–22.3464	–22.3464	–0.8949	–0.8949	–0.8949	–133	–1029	0.44
022	–22.3479	–22.3479	–22.3479	–0.8957	–0.8957	–0.8957	–182	–1026	–0.08
023	–22.3575	–22.3318	–22.3446	–0.8582	–0.9507	–0.9045	–191	–1094	–0.08
024	–22.3586	–22.3586	–22.3586	–0.8685	–0.8685	–0.8685	–721	–1371	–0.86
025	–22.3715	–22.3711	–22.3713	–0.8400	–0.8413	–0.8406	–687	–1318	
026	–22.3269	–22.3327	–22.3298	–0.9411	–0.9249	–0.9330	40	–832	0.69
027	–22.3372	–22.3367	–22.3370	–0.9176	–0.9235	–0.9205	–39	–966	
028	–22.3157	–22.3157	–22.3157	–0.9623	–0.9623	–0.9623	118	–848	0.24
029	–22.3257	–22.3257	–22.3257	–0.9451	–0.9451	–0.9451	103	–774	0.92
030	–22.3280	–22.3280	–22.3280	–0.9436	–0.9436	–0.9436	104	–751	0.92
031	–22.3325	–22.3325	–22.3325	–0.9384	–0.9384	–0.9384	43	–754	0.72
032	–22.3508	–22.3557	–22.3533	–0.8786	–0.8606	–0.8696	–466	–1270	–0.17
033	–22.3523	–22.3570	–22.3547	–0.8838	–0.8567	–0.8702	–508	–1253	–0.20
034	–22.3587	–22.3591	–22.3589	–0.8602	–0.8558	–0.8580	–585	–1299	–0.32
035	–22.3170	–22.3180	–22.3175	–0.9709	–0.9744	–0.9727	199	–755	1.24
036	–22.3611	–22.3578	–22.3594	–0.8595	–0.8650	–0.8622	–597		
037	–22.3565	–22.3565	–22.3565	–0.8646	–0.8646	–0.8646	–543	–1269	–0.34
038	–22.3579	–22.3579	–22.3579	–0.8593	–0.8593	–0.8593	–551	–1299	–0.34
039	–22.3530	–22.3623	–22.3576	–0.8772	–0.8424	–0.8598	–547	–1257	–0.34
040	–22.3618	–22.3654	–22.3636	–0.8542	–0.8403	–0.8472	–640		
041	–22.3622	–22.3671	–22.3647	–0.8536	–0.8345	–0.8440	–619		
042	–22.3564	–22.3547	–22.3555	–0.8648	–0.8757	–0.8702	–518		
043	–22.3672	–22.3672	–22.3672	–0.8490	–0.8490	–0.8490	–685		–0.64

Table S1. *continued*

entry	MEP@O_1	MEP@O_2	MEP@O_{avg}	ΣNAO@O_1	ΣNAO@O_2	ΣNAO@O_{avg}	exp. Q_1	exp. Q_2	exp. σ_p
044	−22.3544	−22.3544	−22.3544	−0.8640	−0.8640	−0.8640	−457	−1259	
045	−22.3601	−22.3601	−22.3601	−0.8618	−0.8618	−0.8618	−581	−1440	−0.40
046	−22.3562	−22.3647	−22.3604	−0.8891	−0.8349	−0.8620	−590	−1363	−0.40
047	−22.3646	−22.3549	−22.3598	−0.8356	−0.8788	−0.8572	−555	−1354	−0.30
048	−22.3602	−22.3602	−22.3602	−0.8551	−0.8550	−0.8551	−649		
049	−22.3587	−22.3629	−22.3608	−0.8632	−0.8467	−0.8550	−632	−1393	−0.51
050	−22.3654	−22.3653	−22.3653	−0.8464	−0.8462	−0.8463	−738	−1390	
051	−22.3592	−22.3585	−22.3589	−0.8630	−0.8687	−0.8658	−500		−0.58
052	−22.3640	−22.3619	−22.3629	−0.8502	−0.8540	−0.8521	−471		
053	−22.3650	−22.3650	−22.3650	−0.8474	−0.8474	−0.8474	−751	−1422	−0.68
054	−22.3666	−22.3686	−22.3676	−0.8547	−0.8524	−0.8536	−810		−0.60
055	−22.3884	−22.3884	−22.3884	−0.7992	−0.7992	−0.7992	−682		
056	−22.3412	−22.3398	−22.3405	−0.8974	−0.9165	−0.9070	−178		0.50
057	−22.3437	−22.3463	−22.3450	−0.8995	−0.8987	−0.8991	−349		0.33
058	−22.3435	−22.3468	−22.3452	−0.8956	−0.8970	−0.8963	−234	−1056	0.33
059	−22.3484	−22.3423	−22.3453	−0.8774	−0.9149	−0.8961	−254		0.33
060	−22.3342	−22.3356	−22.3349	−0.9199	−0.9206	−0.9202	−107	−808	0.73
061	−22.3442	−22.3397	−22.3419	−0.8910	−0.9154	−0.9032	−286	−1116	0.32
062	−22.3506	−22.3573	−22.3539	−0.8830	−0.8558	−0.8694	−392	−1146	−0.01
063	−22.3571	−22.3571	−22.3571	−0.8628	−0.8628	−0.8628	−437	−1146	−0.02
064	−22.3588	−22.3588	−22.3588	−0.8601	−0.8601	−0.8601	−378	−1146	−0.02
065	−22.3444	−22.3481	−22.3463	−0.9007	−0.8810	−0.8908	−372		0.07
066	−22.3363	−22.3355	−22.3359	−0.9120	−0.9221	−0.9171	−40		0.70
067	−22.3317	−22.3399	−22.3358	−0.9361	−0.9022	−0.9191	−50	−744	1.20
068	−22.3322	−22.3369	−22.3345	−0.9327	−0.9182	−0.9254	−41	−752	1.02
069	−22.3374	−22.3430	−22.3402	−0.9141	−0.8940	−0.9040	−58	−727	0.83
070	−22.3442	−22.3435	−22.3438	−0.8962	−0.9068	−0.9015	−147	−1026	0.26
071	−22.3519	−22.3463	−22.3491	−0.8877	−0.8997	−0.8937	−318	−1037	0.19
072	−22.3258	−22.3258	−22.3258	−0.9465	−0.9466	−0.9465	248		1.40
073	−22.3369	−22.3394	−22.3381	−0.9301	−0.9106	−0.9203	−4		0.36
074	−22.3410	−22.3292	−22.3351	−0.9210	−0.9487	−0.9348	−17	−802	0.36
075	−22.3343	−22.3337	−22.3340	−0.9205	−0.9204	−0.9205	−95	−1010	0.54
076	−22.3430	−22.3456	−22.3443	−0.8996	−0.8869	−0.8933	−223	−1018	0.13
077	−22.3447	−22.3474	−22.3461	−0.8945	−0.8821	−0.8883	−207	−1018	
078	−22.3466	−22.3463	−22.3464	−0.8855	−0.8874	−0.8864	−147	−949	
079	−22.3501	−22.3484	−22.3492	−0.8791	−0.8852	−0.8822	−331	−1089	−0.04
080	−22.3499	−22.3477	−22.3488	−0.8808	−0.8859	−0.8834	−293		−0.04
081	−22.3515	−22.3501	−22.3508	−0.8750	−0.8804	−0.8777	−360	−1095	
082	−22.3430	−22.3661	−22.3545	−0.9092	−0.8317	−0.8705	−509	−1213	−0.27
083	−22.3492	−22.3659	−22.3576	−0.8918	−0.8368	−0.8643	−508	−1184	−0.44
084	−22.3605	−22.3564	−22.3585	−0.8560	−0.8553	−0.8557	−458	−1184	−0.54
085	−22.3680	−22.3680	−22.3680	−0.8264	−0.8264	−0.8264	−670	−1282	−0.71
086	−22.3766	−22.3519	−22.3642	−0.8069	−0.8639	−0.8354	−628	−1282	−0.54
087	−22.3631	−22.3630	−22.3631	−0.8533	−0.8373	−0.8453	−539	−1250	−0.71
088	−22.3496	−22.3455	−22.3475	−0.8896	−0.8886	−0.8891	−212	−970	−0.08
089	−22.3673	−22.3639	−22.3656	−0.8434	−0.8411	−0.8422	−645	−1303	
090	−22.3650	−22.3652	−22.3651	−0.8523	−0.8502	−0.8512	−622		

Table S1. ***continued***

entry	MEP@O_1	MEP@O_2	MEP@O_{avg}	ΣNAO@O_1	ΣNAO@O_2	ΣNAO@O_{avg}	exp. Q_1	exp. Q_2	exp. σ_P
091	–22.3657	–22.3684	–22.3670	–0.8354	–0.8436	–0.8395	–611		
092	–22.3666	–22.3652	–22.3659	–0.8502	–0.8364	–0.8433	–611		
093	–22.3638	–22.3623	–22.3630	–0.8585	–0.8455	–0.8520	–602		
094	–22.3509	–22.3572	–22.3541	–0.8827	–0.8547	–0.8687	–766		
095	–22.3509	–22.3572	–22.3541	–0.8827	–0.8547	–0.8687	–441	–1108	–0.07
096	–22.3447	–22.3437	–22.3541	–0.8827	–0.8547	–0.8687	–196	–1044	0.45
097	–22.3327	–22.3361	–22.3442	–0.8920	–0.8915	–0.8917	100	–747	1.15
098	–22.3405	–22.3405	–22.3344	–0.9241	–0.9184	–0.9213	–32		0.90
099	–22.3479	–22.3525	–22.3405	–0.9009	–0.9009	–0.9009	–311	–1184	–0.17
100	–22.3867	–22.3867	–22.3502	–0.8914	–0.8691	–0.8802	–1077	–1674	–1.22
101	–22.3647	–22.3669	–22.3867	–0.7913	–0.7913	–0.7913	–648		
102	–22.3491	–22.3559	–22.3658	–0.8556	–0.8443	–0.8500	–365	–1147	0.02
103	–22.3548	–22.3622	–22.3525	–0.8844	–0.8595	–0.8720	–524	–1241	–0.13
104	–22.3892	–22.3513	–22.3585	–0.8767	–0.8555	–0.8661	–776	–1528	
105	–22.3568	–22.3867	–22.3702	–0.7694	–0.8998	–0.8346	–740	–1418	
106	–22.3514	–22.3899	–22.3717	–0.8708	–0.7922	–0.8315	–746	–1449	
107	–22.3887	–22.3887	–22.3706	–0.9012	–0.7676	–0.8344	–1076	–1614	
108	–22.3348	–22.3582	–22.3887	–0.7879	–0.7879	–0.7879	–390	–1410	–0.54
109	–22.3243	–22.3476	–22.3465	–0.9302	–0.8583	–0.8942	–156	–1060	–0.08
110	–22.3678	–22.3447	–22.3359	–0.9619	–0.8908	–0.9264	–534	–1414	
111	–22.3393	–22.3393	–22.3562	–0.8390	–0.9081	–0.8736	–382		–0.74
112	–22.3230	–22.3230	–22.3393	–0.9221	–0.9221	–0.9221	–144		–0.28
113	–22.3984	–22.3984	–22.3230	–0.9645	–0.9645	–0.9645	–905	–1343	–0.24
114	–22.3671	–22.3580	–22.3984	–0.7620	–0.7620	–0.7620	–475	–1000	
115	–22.3353	–22.3383	–22.3625	–0.8290	–0.8624	–0.8457	–60	–721	0.78

Table S2. Eleven information-theoretic (ITA) descriptors for 1,4-benzoquinones, calculated at the B3LYP/def2-TZVP level. All values are reported in atomic units.

entry	S_S	I_F	S_{GBP}	E_2	E_3	R_2^r	R_3^r	G_1	G_2	G_3	I_G
000	50.124	2974.941	464.395	4.363×10^{2}	2.494×10^{4}	69.839	73.544	–20.933	8.753	78.077	0.936
001	56.293	3559.566	546.985	5.201×10^{2}	2.907×10^{4}	81.909	85.758	–22.898	11.154	89.243	0.969
002	26.755	7922.799	763.700	2.514×10^{3}	1.665×10^{6}	113.772	117.379	–21.724	10.162	94.481	0.888
003	29.229	4572.070	490.189	1.349×10^{3}	8.389×10^{5}	73.662	77.027	–18.400	7.846	69.404	0.843
004	8.770	13024.987	824.541	1.107×10^{4}	7.335×10^{7}	124.964	130.961	–34.523	20.101	115.227	1.506
005	–21.631	12270.629	662.856	1.097×10^{4}	7.335×10^{7}	100.075	104.281	–22.263	10.903	80.504	1.052
006	–68.884	33839.858	702.741	5.509×10^{4}	2.388×10^{9}	134.533	139.629	–30.492	17.376	102.299	1.291
007	–58.866	34090.641	756.579	5.513×10^{4}	2.388×10^{9}	142.823	148.488	–34.286	19.974	114.015	1.443
008	50.124	2974.941	464.395	4.363×10^{2}	2.494×10^{4}	69.839	73.544	–20.933	8.753	78.077	0.936
009	14.429	6753.617	598.514	2.346×10^{3}	1.657×10^{6}	89.744	93.295	–17.905	7.425	72.489	0.879
010	14.486	6753.784	598.569	2.346×10^{3}	1.657×10^{6}	89.546	92.688	–17.731	7.165	71.661	0.780
011	14.488	6753.812	598.567	2.346×10^{3}	1.657×10^{6}	89.625	92.928	–17.763	7.623	71.932	0.820
012	–107.440	21648.682	836.048	2.153×10^{4}	1.467×10^{8}	125.735	129.272	–17.882	7.632	71.252	0.873
013	–242.591	63780.787	700.106	1.096×10^{5}	4.776×10^{9}	161.587	164.808	–17.970	8.354	69.546	0.800
014	54.923	7759.088	814.114	2.473×10^{3}	1.661×10^{6}	122.827	128.536	–33.949	18.847	118.239	1.436
015	–67.043	22653.878	1051.565	2.165×10^{4}	1.467×10^{8}	158.893	164.738	–34.015	17.673	117.584	1.469
016	–202.223	64785.960	915.594	1.098×10^{5}	4.776×10^{9}	194.794	200.431	–34.138	19.292	115.961	1.420
017	34.556	7255.491	706.289	2.409×10^{3}	1.659×10^{6}	106.195	110.611	–25.392	13.253	95.218	1.117
018	95.396	8764.624	1029.640	2.599×10^{3}	1.666×10^{6}	156.034	164.158	–50.186	31.441	164.471	2.054
019	–67.075	22653.682	1051.585	2.165×10^{4}	1.467×10^{8}	158.961	164.944	–33.869	18.574	117.340	1.504
020	–46.749	23157.094	1159.436	2.172×10^{4}	1.467×10^{8}	175.516	182.599	–42.156	25.218	140.180	1.791
021	–29.535	24611.225	1377.916	2.190×10^{4}	1.467×10^{8}	207.747	215.329	–54.341	34.517	176.605	1.902
022	37.579	8155.031	815.771	2.572×10^{3}	1.673×10^{6}	122.636	127.940	–24.795	9.775	113.966	1.344
023	–84.330	23050.073	1053.277	2.175×10^{4}	1.467×10^{8}	158.663	164.032	–24.895	9.993	113.102	1.355
024	–96.194	22326.587	944.464	2.163×10^{4}	1.467×10^{8}	142.060	146.144	–14.830	3.227	92.975	1.063
025	13.200	25327.939	1563.970	2.201×10^{4}	1.467×10^{8}	237.159	247.422	–61.304	38.686	225.982	2.656
026	–0.316	8935.317	706.893	3.343×10^{3}	2.475×10^{6}	105.548	108.713	–17.197	7.198	74.615	0.776
027	–122.200	32787.300	1386.828	3.230×10^{4}	2.200×10^{8}	209.420	216.312	–41.393	25.463	141.028	1.741
028	30.283	4684.140	601.776	8.311×10^{2}	7.974×10^{4}	89.876	93.734	–15.718	–0.841	90.989	0.935
029	–15.121	11116.818	815.217	4.340×10^{3}	3.293×10^{6}	121.503	124.583	–16.591	6.614	77.415	0.749
030	–259.078	40906.386	1290.117	4.270×10^{4}	2.933×10^{8}	193.400	196.261	–15.321	8.750	72.482	0.700
031	–529.472	125170.495	1018.169	2.189×10^{5}	9.552×10^{9}	265.445	268.397	–16.856	7.765	72.674	0.721
032	54.069	2641.423	435.739	3.839×10^{2}	2.188×10^{4}	66.026	70.106	–22.818	10.984	78.269	1.034
033	84.466	3395.786	597.421	4.787×10^{2}	2.522×10^{4}	90.914	96.779	–35.104	20.240	112.985	1.487
034	84.493	3395.714	597.534	4.786×10^{2}	2.521×10^{4}	90.913	96.760	–34.758	20.518	112.443	1.490
035	93.052	5871.543	898.769	9.083×10^{2}	5.934×10^{4}	135.612	142.877	–32.704	10.513	161.657	1.839
036	123.249	4876.514	868.210	6.680×10^{2}	3.191×10^{4}	131.902	139.749	–53.067	34.456	165.482	1.994
037	64.085	2892.163	489.596	4.153×10^{2}	2.298×10^{4}	74.298	78.909	–26.562	14.562	89.995	1.175
038	64.177	2892.547	489.674	4.154×10^{2}	2.298×10^{4}	74.311	78.950	–26.605	14.047	89.694	1.181
039	64.172	2892.556	489.668	4.153×10^{2}	2.299×10^{4}	74.310	78.950	–26.619	14.182	89.702	1.181
040	109.753	4143.512	746.444	5.733×10^{2}	2.855×10^{4}	113.604	120.820	–44.909	28.085	141.673	1.847
041	466.034	13909.433	2747.921	1.806×10^{3}	7.203×10^{4}	419.893	447.681	–189.727	135.365	542.082	7.133
042	102.513	4792.713	801.835	7.043×10^{2}	4.146×10^{4}	121.645	128.918	–40.300	21.303	148.895	1.871
043	124.825	4400.936	813.052	6.051×10^{2}	2.966×10^{4}	124.081	132.243	–50.913	32.332	158.665	2.094
044	71.173	4035.220	639.354	6.091×10^{2}	3.809×10^{4}	96.692	102.078	–28.226	13.783	116.508	1.380
045	124.980	4401.307	813.040	6.051×10^{2}	2.966×10^{4}	124.084	132.292	–51.175	32.729	159.100	2.086

Table S2. *continued*

entry	S_S	I_F	S_{GBP}	E_2	E_3	R_r^2	R_r^3	G_1	G_2	G_3	I_G
046	124.955	4401.253	813.010	6.051×10^{2}	2.967×10^{4}	124.083	132.287	–51.214	32.176	159.162	2.085
047	104.800	3898.878	705.384	5.419×10^{2}	2.744×10^{4}	107.517	114.573	–42.941	26.021	135.316	1.800
048	207.009	6416.813	1245.305	8.581×10^{2}	3.856×10^{4}	190.311	202.911	–82.642	58.184	244.694	3.244
049	74.191	3143.299	543.524	4.470×10^{2}	2.410×10^{4}	82.581	87.748	–30.418	16.917	101.238	1.322
050	272.642	8169.625	1609.751	1.079×10^{3}	4.636×10^{4}	246.321	262.943	–109.394	76.301	321.890	4.275
051	102.232	4791.750	801.783	7.042×10^{2}	4.145×10^{4}	121.671	128.957	–39.537	20.826	148.654	1.894
052	92.715	6717.871	910.139	1.520×10^{3}	6.000×10^{5}	137.815	145.390	–39.803	20.795	153.218	1.967
053	84.201	3394.196	597.370	4.785×10^{2}	2.520×10^{4}	90.863	96.584	–34.272	20.043	112.968	1.469
054	165.420	5407.365	1028.721	7.316×10^{2}	3.411×10^{4}	157.286	167.861	–67.408	44.433	204.528	2.712
055	171.785	6366.695	1164.613	8.573×10^{2}	3.858×10^{4}	177.277	187.847	–73.369	49.014	229.095	2.702
056	60.580	3338.328	531.410	4.967×10^{2}	3.002×10^{4}	80.421	85.292	–25.687	10.956	94.712	1.234
057	70.626	3589.198	585.287	5.283×10^{2}	3.113×10^{4}	88.654	93.973	–29.305	14.235	105.879	1.357
058	70.688	3589.452	585.342	5.282×10^{2}	3.112×10^{4}	88.708	94.144	–29.461	14.267	106.099	1.383
059	70.678	3589.438	585.332	5.282×10^{2}	3.112×10^{4}	88.705	94.136	–29.494	13.965	106.233	1.381
060	45.961	5520.363	639.890	1.494×10^{3}	8.481×10^{5}	96.355	101.108	–24.674	10.968	95.961	1.197
061	91.112	4093.087	693.232	5.915×10^{2}	3.336×10^{4}	105.221	111.683	–37.539	20.594	127.945	1.646
062	82.944	3871.667	652.787	5.415×10^{2}	2.746×10^{4}	98.796	104.449	–37.203	22.011	119.559	1.422
063	121.889	5352.896	923.716	7.307×10^{2}	3.415×10^{4}	139.868	147.689	–55.384	36.167	172.336	1.969
064	121.919	5353.017	923.758	7.308×10^{2}	3.416×10^{4}	139.842	147.617	–55.391	35.874	172.048	1.954
065	94.767	4573.318	761.588	6.543×10^{2}	3.557×10^{4}	115.235	121.754	–40.747	23.133	139.784	1.649
066	75.593	6698.355	870.260	1.518×10^{3}	5.991×10^{5}	131.325	138.003	–37.577	19.121	143.665	1.698
067	92.206	7646.244	1019.835	1.663×10^{3}	6.083×10^{5}	154.014	162.040	–43.943	21.607	171.261	2.055
068	122.547	8400.315	1181.487	1.758×10^{3}	6.117×10^{5}	178.778	188.357	–56.216	31.040	205.688	2.442
069	121.045	8876.448	1236.859	1.820×10^{3}	6.139×10^{5}	186.680	196.085	–58.079	32.286	211.743	2.389
070	95.707	7200.191	978.039	1.582×10^{3}	6.013×10^{5}	147.895	155.693	–45.207	24.108	166.861	1.994
071	105.674	7450.966	1031.840	1.613×10^{3}	6.024×10^{5}	156.188	164.555	–49.152	26.839	178.853	2.147
072	107.243	11006.462	1358.720	2.684×10^{3}	1.177×10^{6}	204.752	214.270	–55.323	30.178	218.809	2.432
073	127.252	11507.917	1466.388	2.748×10^{3}	1.180×10^{6}	221.481	232.429	–63.535	34.453	243.357	2.810
074	127.171	11507.678	1466.330	2.748×10^{3}	1.180×10^{6}	221.493	232.466	–63.337	34.608	243.815	2.816
075	43.914	4363.004	600.663	7.432×10^{2}	6.613×10^{4}	90.143	94.498	–20.561	4.739	95.958	1.082
076	89.557	4568.938	748.558	6.543×10^{2}	3.560×10^{4}	113.109	119.384	–39.630	22.394	134.609	1.582
077	99.636	4820.105	802.446	6.860×10^{2}	3.672×10^{4}	121.279	127.878	–43.118	26.013	144.859	1.673
078	109.760	5071.332	856.359	7.176×10^{2}	3.784×10^{4}	129.728	137.230	–47.585	27.840	157.753	1.901
079	99.611	4819.871	802.439	6.860×10^{2}	3.672×10^{4}	121.375	128.169	–43.391	24.902	145.922	1.722
080	99.664	4820.056	802.489	6.860×10^{2}	3.672×10^{4}	121.387	128.209	–43.439	24.898	145.870	1.727
081	109.681	5071.018	856.318	7.176×10^{2}	3.783×10^{4}	129.647	136.980	–47.291	28.189	157.365	1.862
082	55.552	3091.090	490.470	4.648×10^{2}	2.885×10^{4}	74.183	78.576	–22.406	10.100	87.586	1.114
083	65.610	3342.035	544.357	4.965×10^{2}	2.997×10^{4}	82.457	87.393	–26.261	12.953	99.150	1.257
084	67.203	3792.306	599.122	5.776×10^{2}	3.695×10^{4}	90.658	95.997	–26.143	11.296	108.691	1.359
085	67.212	3791.834	599.135	5.777×10^{2}	3.697×10^{4}	90.645	95.951	–25.718	11.175	108.115	1.355
086	67.222	3791.901	599.141	5.777×10^{2}	3.698×10^{4}	90.644	95.950	–25.732	10.633	107.992	1.353
087	77.306	4043.432	653.048	6.093×10^{2}	3.808×10^{4}	98.942	104.842	–29.983	13.798	119.993	1.506
088	–84.304	23050.343	1053.270	2.175×10^{4}	1.467×10^{8}	158.657	164.015	–25.057	9.924	113.196	1.351
089	87.320	4294.165	706.901	6.408×10^{2}	3.918×10^{4}	107.214	113.644	–33.783	16.774	131.640	1.647
090	179.189	6560.153	1192.771	9.254×10^{2}	4.919×10^{4}	181.894	193.668	–70.104	44.487	231.804	3.029
091	122.872	5294.478	909.801	7.672×10^{2}	4.364×10^{4}	138.223	146.676	–48.398	27.765	172.229	2.165
092	194.032	7295.106	1315.672	1.020×10^{3}	5.256×10^{4}	200.136	212.408	–77.141	49.327	252.008	3.148

Table S2. ***continued***

entry	S_S	I_F	S_{GBP}	E_2	E_3	R_r^2	R_r^3	G_1	G_2	G_3	I_G
093	442.993	14296.816	2736.126	1.905×10^3	8.379×10^4	417.283	443.861	−178.818	124.261	534.949	6.814
094	437.059	13935.268	2681.779	7.564×10^2	4.453×10^4	120.538	125.602	−36.171	23.177	137.949	1.302
095	65.636	3342.760	544.429	4.965×10^2	2.998×10^4	82.476	87.424	−25.985	12.848	98.381	1.272
096	62.100	3788.204	586.176	5.777×10^2	3.701×10^4	88.554	93.685	−25.042	10.013	103.467	1.304
097	93.680	8095.834	1074.576	1.744×10^3	6.153×10^5	162.072	170.226	−43.494	20.367	180.066	2.084
098	80.240	5186.108	790.539	8.034×10^2	5.326×10^4	119.380	126.151	−31.163	11.059	140.176	1.729
099	87.081	5109.061	803.321	7.564×10^2	4.453×10^4	121.077	127.303	−35.515	17.393	143.444	1.563
100	96.192	4076.327	706.229	5.830×10^2	3.114×10^4	107.205	113.561	−33.001	17.578	133.809	1.656
101	111.359	11015.402	1330.507	2.970×10^3	1.692×10^6	200.787	210.256	−42.613	19.269	215.484	2.474
102	73.489	5797.867	761.228	1.357×10^3	5.859×10^5	114.873	120.685	−37.406	22.498	123.888	1.461
103	105.423	6076.258	867.614	1.389×10^3	5.871×10^5	131.911	139.769	−47.435	29.224	151.720	1.998
104	120.760	4490.045	813.562	6.257×10^2	3.152×10^4	123.932	131.796	−45.982	27.772	157.870	2.017
105	130.891	4741.610	867.427	6.574×10^2	3.263×10^4	132.213	140.644	−50.258	31.083	169.519	2.159
106	130.916	4741.873	867.435	6.573×10^2	3.263×10^4	132.216	140.656	−50.360	31.348	169.537	2.161
107	136.661	5080.920	921.741	7.096×10^2	3.563×10^4	140.339	148.980	−48.648	28.418	179.949	2.233
108	55.344	3089.900	490.432	4.650×10^2	2.887×10^4	74.141	78.407	−21.512	9.007	87.199	1.101
109	−96.256	22347.490	944.537	2.164×10^4	1.467×10^8	142.102	146.325	−20.269	8.165	92.286	1.071
110	177.481	6109.225	1138.008	8.444×10^2	4.222×10^4	173.706	185.073	−69.661	44.898	222.206	2.939
111	46.628	3287.696	491.298	5.145×10^2	3.475×10^4	73.982	77.887	−16.374	4.363	84.382	1.031
112	17.066	7650.805	707.930	2.509×10^3	1.671×10^6	105.795	109.361	−15.275	3.457	89.823	0.922
113	144.481	5568.024	1001.969	7.722×10^2	3.782×10^4	152.736	162.190	−55.452	34.488	199.507	2.437
114	97.684	4699.110	788.320	6.566×10^2	3.266×10^4	119.126	125.398	−40.926	24.759	146.063	1.601
115	47.409	5970.060	694.602	1.575×10^3	8.551×10^5	104.522	109.598	−24.367	9.290	105.552	1.284

Table S3. Molecular electrostatic potential on two oxygen atom nuclei (MEP@O), sum of valence NAO energies of two oxygen atoms (ΣNAO@O), and the experimental 1st/2nd redox potential (Q_1/Q_2, in mV) and Hammett parameters (σ_P), for a total of (108 out of 110) 9,10-anthraquinone derivatives, calculated at the B3LYP/def2-TZVP level of theory.

entry	MEP@O_1	MEP@O_2	MEP@O_{avg}	ΣNAO@O_1	ΣNAO@O_2	ΣNAO@O_{avg}	exp. Q_1	exp. Q_2	exp. σ_P
117	–22.3668	–22.3641	–22.3654	–0.8452	–0.8515	–0.8483	–746	–1447	0.23
118	–22.3610	–22.3629	–22.3620	–0.8582	–0.8533	–0.8558	–732	–1452	0.23
119	–22.3612	–22.3627	–22.3619	–0.8577	–0.8538	–0.8558	–700	–1501	0.18
120	–22.3646	–22.3649	–22.3647	–0.8478	–0.8478	–0.8478	–764	–1435	0.06
121	–22.3634	–22.3634	–22.3634	–0.8565	–0.8565	–0.8565	–719	–1444	0.46
122	–22.3664	–22.3608	–22.3636	–0.8500	–0.8625	–0.8562	–717	–1445	0.46
123	–22.3711	–22.3708	–22.3709	–0.8382	–0.8321	–0.8351	–867	–1494	–0.17
124	–22.3697	–22.3711	–22.3704	–0.8340	–0.8299	–0.8319	–853	–1470	–0.17
125	–22.3699	–22.3714	–22.3706	–0.8335	–0.8292	–0.8313	–849	–1487	–0.15
126	–22.3706	–22.3717	–22.3711	–0.8317	–0.8279	–0.8298	–852	–1470	–0.20
127	–22.3746	–22.3746	–22.3746	–0.8297	–0.8297	–0.8297	–944	–1589	–0.34
128	–22.3739	–22.3747	–22.3743	–0.8243	–0.8364	–0.8303	–964	–1558	–0.34
129	–22.3730	–22.3730	–22.3730	–0.8246	–0.8246	–0.8246	–885	–1580	–0.34
130	–22.3717	–22.3745	–22.3731	–0.8283	–0.8202	–0.8243	–897	–1486	–0.34
131	–22.3795	–22.3795	–22.3795	–0.8154	–0.8154	–0.8154	–1009	–1674	–0.68
132	–22.3781	–22.3781	–22.3781	–0.8100	–0.8100	–0.8100	–961	–1510	–0.68
133	–22.3690	–22.3681	–22.3685	–0.8473	–0.8390	–0.8432	–672	–1355	
134	–22.3734	–22.3721	–22.3728	–0.8350	–0.8276	–0.8313	–761	–1397	
135	–22.3706	–22.3725	–22.3716	–0.8340	–0.8318	–0.8329	–873	–1488	
136	–22.3832	–22.3861	–22.3847	–0.8011	–0.7921	–0.7966	–1036	–1491	
137	–22.3808	–22.3734	–22.3771	–0.8011	–0.8244	–0.8128	–900	–1418	–0.27
138	–22.3708	–22.3759	–22.3734	–0.8303	–0.8173	–0.8238	–870	–1462	–0.27
139	–22.3740	–22.3740	–22.3740	–0.8274	–0.8274	–0.8274	–952		–0.54
140	–22.3863	–22.3863	–22.3863	–0.7868	–0.7868	–0.7868	–956		–0.54
141	–22.3791	–22.3791	–22.3791	–0.8076	–0.8076	–0.8076	–904	–1490	–0.54
142	–22.3737	–22.3834	–22.3785	–0.8214	–0.7969	–0.8092	–874	–1427	–0.54
143	–22.3600	–22.3602	–22.3601	–0.8677	–0.8620	–0.8648	–772	–1218	0.31
144	–22.3561	–22.2830	–22.3195	–0.8922	–1.0772	–0.9847	–768	–1410	0.31
145	–22.3541	–22.3541	–22.3541	–0.8859	–0.8859	–0.8859	–728	–1197	0.62
146	–22.3554	–22.3541	–22.3548	–0.8889	–0.8807	–0.8848	–723	–1128	0.62
147	–22.3672	–22.3672	–22.3672	–0.8409	–0.8409	–0.8409	–742	–1380	0.62
148	–22.3636	–22.3704	–22.3670	–0.8513	–0.8316	–0.8414	–726	–1380	0.62
149	–22.3752	–22.3717	–22.3734	–0.8212	–0.8291	–0.8251	–799	–1449	
150	–22.3723	–22.3764	–22.3744	–0.8430	–0.8153	–0.8291	–942	–1610	–0.66
151	–22.3736	–22.3829	–22.3782	–0.8239	–0.7974	–0.8106	–1019		–0.66
152	–22.3693	–22.3756	–22.3725	–0.8533	–0.8205	–0.8369	–866	–1495	–0.43
153	–22.3717	–22.3728	–22.3722	–0.8492	–0.8275	–0.8383	–865	–1499	–0.43
154	–22.3739	–22.3796	–22.3767	–0.8392	–0.8063	–0.8228	–971		–0.83
155	–22.3780	–22.3893	–22.3836	–0.8279	–0.7776	–0.8028	–983	–1483	–0.93
156	–22.3868	–22.3868	–22.3868	–0.8025	–0.8030	–0.8028	–1070	–1558	–1.50
157	–22.4695	–22.4740	–22.4717	–0.5435	–0.5421	–0.5428	–487	–921	
158	–22.3635	–22.3671	–22.3653	–0.8678	–0.8416	–0.8547	–772	–1384	–0.20
159	–22.3621	–22.3697	–22.3659	–0.8750	–0.8397	–0.8573	–754		–0.20
160	–22.3677	–22.3735	–22.3706	–0.8448	–0.8252	–0.8350	–869	–1547	–0.20

Table S3. *continued*

entry	MEP@O1	MEP@O2	MEP@Oavg	ΣNAO@O1	ΣNAO@O2	ΣNAO@Oavg	exp. Q1	exp. Q2	exp. σ_p
161	–22.3667	–22.3733	–22.3700	–0.8502	–0.8258	–0.8380	–872	–1518	–0.20
162	–22.3709	–22.3792	–22.3750	–0.8498	–0.8104	–0.8301	–930	–1529	–0.60
163	–22.4698	–22.4668	–22.4683	–0.5562	–0.5553	–0.5557	–835	–1486	
164	–22.3574	–22.3492	–22.3533	–0.8872	–0.9105	–0.8989	–938		
165	–22.3739	–22.3859	–22.3799	–0.8380	–0.7892	–0.8136	−1156	−1671	–1.32
166	–22.3865	–22.3865	–22.3865	–0.8032	–0.8032	–0.8032	−1112	−1605	–1.32
167	–22.3805	–22.3805	–22.3805	–0.8200	–0.8200	–0.8200	−1031	−1618	–1.32
168	–22.3886	–22.3886	–22.3886	–0.7819	–0.7819	–0.7819	−1219		–1.32
169	–22.3974	–22.3974	–22.3974	–0.7909	–0.7909	–0.7909	−1321	−1707	–2.64
170	–22.3726	–22.3770	–22.3748	–0.8425	–0.8137	–0.8281	−899	−1520	–0.84
171	–22.3852	–22.3843	–22.3848	–0.8076	–0.8105	–0.8091	−1009		
172	–22.3869	–22.3869	–22.3869	–0.8026	–0.8026	–0.8026	−1010		–1.68
173	–22.3900	–22.3900	–22.3900	–0.7943	–0.7943	–0.7943	−1040	−1618	
174	–22.3537	–22.3534	–22.3536	–0.8792	–0.8809	–0.8800	−581		0.78
175	–22.3562	–22.3579	–22.3571	–0.8734	–0.8678	–0.8706	−615		0.61
176	–22.3751	–22.3745	–22.3748	–0.8375	–0.8386	–0.8380	−929		–0.54
177	–22.3517	–22.3662	–22.3590	–0.9004	–0.8434	–0.8719	−632	−1248	–0.37
178	–22.3687	–22.3736	–22.3711	–0.8363	–0.8239	–0.8301	−814	−1316	–0.37
179	–22.3579	–22.3814	–22.3697	–0.8840	–0.7991	–0.8415	−720		–0.64
180	–22.3716	–22.3636	–22.3676	–0.8263	–0.8580	–0.8422	−606	−1214	–0.06
181	–22.3645	–22.3765	–22.3705	–0.8645	–0.8307	–0.8476	−845	−1420	–1.03
182	–22.3634	–22.3731	–22.3682	–0.8680	–0.8413	–0.8546	−679	−1235	
183	–22.3587	–22.3692	–22.3639	–0.8811	–0.8521	–0.8666	−682	−1244	–0.80
184	–22.3480	–22.3727	–22.3604	–0.9112	–0.8255	–0.8684	−683	−1271	–0.74
185	–22.3546	–22.3546	–22.3546	–0.8920	–0.8920	–0.8920	−558	−1188	–0.74
186	–22.3505	–22.3505	–22.3505	–0.9036	–0.9036	–0.9036	−492	−989	–0.74
187	–22.3370	–22.3656	–22.3513	–0.9602	–0.8453	–0.9028	−521	−1206	–0.74
188	–22.3748	–22.3748	–22.3748	–0.8197	–0.8197	–0.8197	−879		–0.74
189	–22.3696	–22.3790	–22.3743	–0.8329	–0.8094	–0.8212	−862		–0.74
190	–22.4730	–22.4756	–22.4743	–0.5286	–0.5241	–0.5264	−717		
191	–22.3602	–22.3660	–22.3631	–0.8770	–0.8582	–0.8676	−614	−1202	1.01
192	–22.3618	–22.3669	–22.3644	–0.8721	–0.8569	–0.8645	−665		
193	–22.3605	–22.3483	–22.3544	–0.8749	–0.9273	–0.9011	−638		
194	–22.4543	–22.4517	–22.4530	–0.5984	-0.6076	-0.6030	−562	−1019	
195	–22.3394	–22.3406	–22.3400	–0.9362	–0.9312	–0.9337	−248	−788	–0.01
196	–22.3411	–22.3677	–22.3544	–0.9483	–0.8395	–0.8939	−559	−1212	–0.91
197	–22.3609	–22.3608	–22.3609	–0.8743	–0.8743	–0.8743	−640	−1269	–1.08
198	–22.3589	–22.3801	–22.3695	–0.8994	–0.8383	–0.8688	−813	−1375	–2.06
199	–22.3538	–22.3712	–22.3625	–0.8950	–0.8289	–0.8620	−720		–1.11
200	–22.3493	–22.3615	–22.3554	–0.9072	–0.8725	–0.8898	−619	−1201	–1.11
201	–22.3536	–22.3395	–22.3465	–0.8948	–0.9529	–0.9238	−426	−1015	–1.11
202	–22.3598	–22.3558	–22.3578	–0.8778	–0.8879	–0.8829	−610	−1209	–1.11
203	–22.3462	–22.3680	–22.3571	–0.9342	–0.8380	–0.8861	−568	−1104	–1.28
204	–22.3363	–22.3589	–22.3476	–0.9621	–0.8799	–0.9210	−536	−1110	–1.48
205	–22.3415	–22.3415	–22.3415	–0.9472	–0.9472	–0.9472	−381	−942	–1.48
206	–22.3432	–22.3457	–22.3444	–0.9428	–0.9348	–0.9388	−424	−975	–1.65
207	–22.3371	–22.3472	–22.3421	–0.9600	–0.9309	–0.9455	−475	−1044	–1.85

Table S3. ***continued***

entry	MEP@O1	MEP@O2	MEP@Oavg	ΣNAO@O1	ΣNAO@O2	ΣNAO@Oavg	exp. Q1	exp. Q2	exp. σ_p
208	–22.3565	–22.3565	–22.3565	–0.8864	–0.8864	–0.8864	−495		–2.22
211	–22.3715	–22.3693	–22.3704	–0.8292	–0.8474	–0.8383	−717		
212	–22.3814	–22.3769	–22.3792	–0.8144	–0.8149	–0.8146	−925	−1550	
213	–22.3756	–22.3845	–22.3801	–0.8183	–0.7922	–0.8052	−912	−1526	
214	–22.3685	–22.3697	–22.3691	–0.8374	–0.8339	–0.8357	−830	−1542	
215	–22.3570	–22.3557	–22.3563	–0.8683	–0.8736	–0.8710	–598	–1285	0.90
216	–22.3459	–22.3459	–22.3459	–0.8996	–0.8996	–0.8996	–398	–1082	1.80
217	–22.3472	–22.3446	–22.3459	–0.8943	–0.9047	–0.8995	–394	–1065	1.80
218	–22.3581	–22.3569	–22.3575	–0.8657	–0.8700	–0.8678	–682		0.57
219	–22.3481	–22.3481	–22.3481	–0.8937	–0.8937	–0.8937	–547	–1056	1.14
220	–22.3494	–22.3469	–22.3482	–0.8893	–0.8978	–0.8935	–538		1.14
221	–22.3646	–22.3621	–22.3633	–0.8464	–0.8552	–0.8508	–691	–1400	0.45
222	–22.4850	–22.4656	–22.4753	-0.4780	–0.5563	–0.5171	–861		
223	–22.4601	–22.4589	–22.4595	–0.5645	–0.5730	–0.5687	–834	–1472	
224	–22.5759	–22.5759	–22.5759	–0.2159	–0.2159	–0.2159	–872		
225	–22.5444	–22.5444	–22.5444	–0.3175	–0.3175	–0.3175	–833	–1446	
226	–22.5468	–22.5424	–22.5446	–0.3058	–0.3280	–0.3169	–830	–1404	

Table S4. Molecular electrostatic potential on two oxygen atom nuclei (MEP@O), sum of valence NAO energies of two oxygen atoms (ΣNAO@O), and the experimental 1st/2nd redox potential (Q_1/Q_2, in mV) and Hammett parameters (σ_P), for a total of 90 1,4-naphthoquinone derivatives, calculated at the B3LYP/def2-TZVP level of theory.

entry	MEP@O_1	MEP@O_2	MEP@O_{avg}	ΣNAO@O_1	ΣNAO@O_2	ΣNAO@O_{avg}	exp. Q_1	exp. Q_2	exp. σ_P
227	−22.3418	−22.3412	−22.3415	−0.9052	−0.9071	−0.9061	−387	−1116	0.66
228	−22.3507	−22.3491	−22.3499	−0.8859	−0.8926	−0.8892	−523	−1252	0.32
229	−22.3549	−22.3487	−22.3518	−0.8686	−0.8862	−0.8774	−402	−1199	0.23
230	−22.3487	−22.3821	−22.3654	−0.9007	−0.7951	−0.8479	−704	−1382	
231	−22.3474	−22.3474	−22.3474	−0.8905	−0.8905	−0.8905	−287	−1238	0.46
232	−22.3482	−22.3482	−22.3482	−0.8872	−0.8872	−0.8872	−424	−1154	0.46
233	−22.3479	−22.3479	−22.3479	−0.8917	−0.8917	−0.8917	−292	−1087	0.46
234	−22.3550	−22.3497	−22.3524	−0.8684	−0.8878	−0.8781	−500	−1227	0.29
235	−22.3554	−22.3554	−22.3554	−0.8738	−0.8738	−0.8738	−577	−1274	0.12
236	−22.3652	−22.3600	−22.3626	−0.8397	−0.8589	−0.8493	−650	−1384	−0.17
237	−22.3625	−22.3624	−22.3625	−0.8545	−0.8488	−0.8517	−636	−1382	−0.17
238	−22.3609	−22.3623	−22.3616	−0.8521	−0.8478	−0.8499	−593	−1395	−0.17
239	−22.3662	−22.3614	−22.3638	−0.8365	−0.8656	−0.8511	−662	−1418	−0.20
240	−22.3680	−22.3619	−22.3649	−0.8322	−0.8572	−0.8447	−679	−1418	−0.22
241	−22.3656	−22.3656	−22.3656	−0.8454	−0.8454	−0.8454	−746	−1457	−0.34
242	−22.3676	−22.3639	−22.3658	−0.8333	−0.8481	−0.8407	−729	−1440	−0.34
243	−22.3688	−22.3624	−22.3656	−0.8296	−0.8524	−0.8410	−674	−1368	−0.34
244	−22.3690	−22.3641	−22.3666	−0.8305	−0.8554	−0.8430	−710		−0.34
245	−22.3669	−22.3669	−22.3669	−0.8439	−0.8439	−0.8439	−719	−1328	−0.34
246	−22.3645	−22.3645	−22.3645	−0.8418	−0.8418	−0.8418	−622	−1408	−0.34
247	−22.3665	−22.3669	−22.3667	−0.8444	−0.8411	−0.8428	−744	−1440	−0.32
248	−22.3670	−22.3675	−22.3673	−0.8430	−0.8392	−0.8411	−748	−1440	−0.32
249	−22.3673	−22.3680	−22.3676	−0.8420	−0.8378	−0.8399	−756	−1449	
250	−22.3673	−22.3680	−22.3676	−0.8420	−0.8378	−0.8399	−730	−1440	
251	−22.3671	−22.3670	−22.3671	−0.8404	−0.8427	−0.8416	−709		
252	−22.3657	−22.3676	−22.3667	−0.8497	−0.8389	−0.8443	−705		
253	−22.3660	−22.3680	−22.3670	−0.8488	−0.8378	−0.8433	−709		
254	−22.3547	−22.3546	−22.3547	−0.8698	−0.8704	−0.8701	−567		
255	−22.3705	−22.3694	−22.3699	−0.8385	−0.8368	−0.8376	−840	−1597	−0.51
256	−22.3711	−22.3711	−22.3711	−0.8298	−0.8298	−0.8298	−781		−0.68
257	−22.3737	−22.3596	−22.3667	−0.8188	−0.8522	−0.8355	−737	−1415	−0.27
258	−22.3623	−22.3609	−22.3616	−0.8532	−0.8531	−0.8531	−683		−0.27
259	−22.3786	−22.3607	−22.3696	−0.8058	−0.8483	−0.8271	−695		−0.44
260	−22.3661	−22.3696	−22.3679	−0.8344	−0.8371	−0.8357	−653	−1382	−0.54
261	−22.3804	−22.3804	−22.3804	−0.7960	−0.7960	−0.7960	−776		−0.54
262	−22.3504	−22.3508	−22.3506	−0.8870	−0.8816	−0.8843	−547		0.31
263	−22.3500	−22.3366	−22.3433	−0.8878	−0.9378	−0.9128	−356	−1023	−0.06
264	−22.3446	−22.3415	−22.3431	−0.9039	−0.9123	−0.9081	−509	−1155	0.62
265	−22.3470	−22.3403	−22.3436	−0.8985	−0.9200	−0.9092	−386	−1138	0.93
266	−22.3742	−22.3601	−22.3671	−0.8174	−0.8508	−0.8341	−700	−1354	−0.25
267	−22.3772	−22.3621	−22.3697	−0.8093	−0.8455	−0.8274	−701	−1399	−0.45
268	−22.3472	−22.3464	−22.3468	−0.8877	−0.8987	−0.8932	−167	−940	0.70
269	−22.3866	−22.3529	−22.3698	−0.7828	−0.8850	−0.8339	−845	−1499	−0.66
270	−22.3694	−22.3548	−22.3621	−0.8394	−0.8786	−0.8590	−710	−1364	−0.51

Table S4. *continued*

entry	MEP@O1	MEP@O2	MEP@Oavg	ΣNAO@O1	ΣNAO@O2	ΣNAO@Oavg	exp. Q1	exp. Q2	exp. σ_p
271	–22.3714	–22.3542	–22.3628	–0.8387	–0.8811	–0.8599	–767		–0.66
272	–22.3717	–22.3550	–22.3634	–0.8375	–0.8789	–0.8582	–771	–1386	
273	–22.3870	–22.3564	–22.3717	–0.7881	–0.8731	–0.8306	–861	–1593	–1.49
274	–22.3892	–22.3604	–22.3748	–0.7747	–0.8699	–0.8223	–845	–1456	–0.83
275	–22.3476	–22.3785	–22.3630	–0.9039	–0.8016	–0.8528	–704	–1365	
276	–22.3709	–22.3565	–22.3637	–0.8255	–0.8689	–0.8472	–619	–1346	0.00
277	–22.3666	–22.3639	–22.3653	–0.8433	–0.8479	–0.8456	–651	–1380	–0.17
278	–22.3676	–22.3650	–22.3663	–0.8405	–0.8445	–0.8425	–641	–1414	–0.14
279	–22.3691	–22.3375	–22.3533	–0.8304	–0.9282	–0.8793	–460	–1390	–0.37
280	–22.3437	–22.3570	–22.3504	–0.9160	–0.8624	–0.8892	–390	–1078	–0.37
281	–22.3691	–22.3439	–22.3565	–0.8349	–0.9096	–0.8723	–582	–1420	–0.54
282	–22.3698	–22.3438	–22.3568	–0.8332	–0.9105	–0.8718	–590	–1469	–0.52
283	–22.3702	–22.3443	–22.3572	–0.8321	–0.9088	–0.8705	–573	–1444	–0.52
284	–22.3444	–22.3702	–22.3573	–0.9087	–0.8319	–0.8703	–594	–1475	
285	–22.3444	–22.3702	–22.3573	–0.9086	–0.8319	–0.8702	–594	–1491	
286	–22.3444	–22.3703	–22.3573	–0.9085	–0.8318	–0.8701	–573	–1448	
287	–22.3445	–22.3703	–22.3574	–0.9085	–0.8318	–0.8701	–603	–1451	
288	–22.3445	–22.3703	–22.3574	–0.9084	–0.8317	–0.8700	–770	–1452	
289	–22.3701	–22.3444	–22.3572	–0.8322	–0.9087	–0.8704	–604	–1449	–0.60
290	–22.3693	–22.3444	–22.3568	–0.8364	–0.9084	–0.8724	–606	–1502	
291	–22.3446	–22.3703	–22.3574	–0.9081	–0.8317	–0.8699	–606	–1497	
292	–22.3686	–22.3430	–22.3558	–0.8367	–0.9125	–0.8746	–599	–1452	
293	–22.3682	–22.3426	–22.3554	–0.8380	–0.9140	–0.8760	–607	–1435	
294	–22.3696	–22.3440	–22.3568	–0.8338	–0.9098	–0.8718	–573	–1444	
295	–22.3430	–22.3688	–22.3559	–0.9127	–0.8364	–0.8745	–582	–1461	
296	–22.3701	–22.3447	–22.3574	–0.8333	–0.9081	–0.8707	–597	–1501	
297	–22.3666	–22.3469	–22.3567	–0.8402	–0.9019	–0.8710	–369	–1320	
298	–22.3444	–22.3700	–22.3572	–0.9086	–0.8325	–0.8706	–582	–1429	
299	–22.3444	–22.3700	–22.3572	–0.9086	–0.8326	–0.8706	–616	–1444	
300	–22.3352	–22.3440	–22.3396	–0.9096	–0.9099	–0.9097	–510	–1444	
301	–22.3687	–22.3399	–22.3543	–0.8289	–0.9222	–0.8755	–446	–1299	
302	–22.3410	–22.3672	–22.3541	–0.9186	–0.8441	–0.8814	–790	–1446	
303	–22.3666	–22.3417	–22.3542	–0.8448	–0.9162	–0.8805	–782	–1429	
304	–22.3763	–22.3410	–22.3587	–0.8106	–0.9174	–0.8640	–475	–1397	–0.64
305	–22.3771	–22.3480	–22.3625	–0.8132	–0.8979	–0.8555	–636	–1507	
306	–22.3352	–22.3536	–22.3444	–0.9423	–0.8722	–0.9073	–233	–936	–0.14
307	–22.3499	–22.3584	–22.3541	–0.8987	–0.8633	–0.8810	–479	–1176	–0.54
308	–22.3453	–22.3639	–22.3546	–0.9165	–0.8433	–0.8799	–464	–1117	–0.54
309	–22.3617	–22.3493	–22.3555	–0.8444	–0.8938	–0.8691	–590	–1331	–0.74
310	–22.3479	–22.3479	–22.3479	–0.9041	–0.9041	–0.9041	–284	–979	–0.74
311	–22.3459	–22.3747	–22.3603	–0.9037	–0.8199	–0.8618	–667		
312	–22.3410	–22.3588	–22.3499	–0.9342	–0.8801	–0.9071	–527		
313	–22.3731	–22.3587	–22.3659	–0.8184	–0.8623	–0.8403	–496	–1219	
314	–22.3655	–22.3631	–22.3643	–0.8455	–0.8499	–0.8477	–521	–1269	
315	–22.4755	–22.4693	–22.4724	–0.5054	–0.5354	–0.5204	–584		
316	–22.3525	–22.3525	–22.3525	–0.8765	–0.8765	–0.8765	–1016		

Table S5. Molecular electrostatic potential on two oxygen atom nuclei (MEP@O), sum of valence NAO energies of two oxygen atoms (ΣNAO@O), and the experimental 1st/2nd redox potential (Q_1/Q_2, in mV), for a total of 33 miscellaneous quinone and nonquinone derivatives, calculated at the B3LYP/def2-TZVP level of theory.

entry	MEP@O_1	MEP@O_2	MEP@O_{avg}	ΣNAO@O_1	ΣNAO@O_2	ΣNAO@O_{avg}	exp. Q_1	exp. Q_2
317	–22.3281	–22.3281	–22.3281	–0.9277	–0.9277	–0.9277	197	–645
318	–22.3304	–22.3304	–22.3304	–0.9238	–0.9238	–0.9238	201	–602
319	–22.3589	–22.3669	–22.3629	–0.8481	–0.8217	–0.8349	–458	–1142
320	–22.3663	–22.3771	–22.3717	–0.8214	–0.7931	–0.8072	–365	–827
321	–22.3492	–22.3500	–22.3496	–0.8769	–0.8692	–0.8730	–326	–1254
322	–22.3640	–22.3786	–22.3713	–0.8346	–0.7928	–0.8137	–480	–1406
323	–22.3683	–22.3855	–22.3769	–0.8227	–0.7728	–0.7978	–750	–1414
324	–22.3704	–22.3954	–22.3829	–0.8168	–0.7465	–0.7817	–759	–1461
325	–22.3600	–22.3750	–22.3675	–0.8458	–0.8029	–0.8243	–410	–1061
326	–22.4611	–22.4737	–22.4674	–0.5530	–0.5111	–0.5320	–433	–1040
327	–22.5539	–22.5600	–22.5570	–0.2826	–0.2607	–0.2717	–404	
328	–22.3691	–22.3827	–22.3759	–0.8212	–0.7819	–0.8016	–458	–1401
329	–22.3762	–22.3434	–22.3598	–0.8118	–0.9122	–0.8620	–487	–1367
330	–22.3707	–22.3697	–22.3702	–0.8245	–0.8202	–0.8223	–670	–1257
331	–22.3616	–22.3616	–22.3616	–0.8437	–0.8437	–0.8437	–624	
332	–22.3781	–22.3781	–22.3781	–0.8054	–0.8054	–0.8054	–331	–704
333	–22.3843	–22.3843	–22.3843	–0.7844	–0.7844	–0.7844	–314	–712
334	–22.3857	–22.3857	–22.3857	–0.7752	–0.7752	–0.7752	–331	–700
335	–22.3887	–22.2810	–22.3348	–0.7811	–1.0529	–0.9170	–1603	
336	–22.3673	–22.2661	–22.3167	–0.8546	–1.1022	–0.9784	–1500	
337	–22.3887			–0.7881			–1540	
338	–22.3676	–22.3919	–22.3797	–0.8423	–0.8579	–0.8501	–1121	
339	–22.3586	–22.3684	–22.3635	–0.8703	–0.9326	–0.9015	–971	
340	–22.3511	–22.3352	–22.3431	–0.9231	–0.9497	–0.9364	–1310	
341	–22.3890			–0.7831			–1645	
342	–22.4792	–22.5657	–22.5224	–0.5141	–0.3649	-0.4395	–1687	
343	–22.3710			–0.8179			–1223	
344	–22.3713			–0.8422			–1642	
345	–22.3994	–22.2767	–22.3380	–0.7486	–1.0725	–0.9105	–1618	
346	–22.3609	–22.3609	–22.3609	–0.8631	–0.8631	–0.8631	–1067	
347							353	–268
348	–22.3683	–22.3676	–22.3679	–0.8443	–0.8343	–0.8393	–759	–1503
349							–1104	

Table S6. Eleven information-theoretic (ITA) descriptors for 9,10-anthraquinone derivatives, calculated at the B3LYP/def2-TZVP level. All values are reported in atomic units.

entry	S_S	I_F	S_{GBP}	E_2	E_3	R_2^r	R_3^r	G_1	G_2	G_3	I_G
117	76.630	6539.750	842.037	1.602×10^{3}	8.479×10^{5}	1.265×10^{2}	1.317×10^{2}	–41.382	28.663	135.146	1.3015
118	76.703	6539.946	842.110	1.602×10^{3}	8.478×10^{5}	1.266×10^{2}	1.317×10^{2}	–41.395	27.388	134.934	1.3086
119	–51.807	35053.404	892.879	5.525×10^{4}	2.388×10^{9}	1.629×10^{2}	1.688×10^{2}	–41.398	26.051	137.582	1.4757
120	25.840	14238.512	1014.750	1.122×10^{4}	7.336×10^{7}	1.528×10^{2}	1.586×10^{2}	–45.254	30.297	145.961	1.4551
121	61.872	8721.453	950.368	2.599×10^{3}	1.666×10^{6}	1.425×10^{2}	1.474×10^{2}	–40.972	29.153	137.728	1.2619
122	61.876	8721.461	950.369	2.599×10^{3}	1.666×10^{6}	1.425×10^{2}	1.474×10^{2}	–40.934	28.948	137.455	1.2569
123	101.453	4609.158	787.575	6.362×10^{2}	3.082×10^{4}	1.189×10^{2}	1.247×10^{2}	–45.821	30.931	144.234	1.4874
124	101.567	4609.453	787.681	6.363×10^{2}	3.082×10^{4}	1.189×10^{2}	1.248×10^{2}	–45.716	30.382	143.793	1.4952
125	111.782	4861.359	841.665	6.677×10^{2}	3.191×10^{4}	1.272×10^{2}	1.337×10^{2}	–49.787	35.464	154.950	1.6553
126	131.998	5363.948	949.399	7.309×10^{2}	3.414×10^{4}	1.438×10^{2}	1.515×10^{2}	–57.894	41.200	178.192	1.9538
127	111.514	4860.275	841.436	6.676×10^{2}	3.191×10^{4}	1.272×10^{2}	1.336×10^{2}	–49.791	35.491	155.859	1.6300
128	111.520	4860.288	841.441	6.676×10^{2}	3.191×10^{4}	1.272×10^{2}	1.336×10^{2}	–49.791	35.022	155.781	1.6302
129	111.653	4860.582	841.587	6.676×10^{2}	3.191×10^{4}	1.272×10^{2}	1.336×10^{2}	–49.505	34.049	155.164	1.6459
130	111.733	4860.847	841.655	6.676×10^{2}	3.191×10^{4}	1.272×10^{2}	1.337×10^{2}	–49.487	35.320	154.921	1.6471
131	131.843	5363.031	949.383	7.308×10^{2}	3.413×10^{4}	1.438×10^{2}	1.513×10^{2}	–57.475	41.124	178.153	1.9342
132	131.918	5363.132	949.467	7.308×10^{2}	3.414×10^{4}	1.438×10^{2}	1.514×10^{2}	–57.224	39.671	177.773	1.9483
133	115.203	5342.301	909.664	7.307×10^{2}	3.416×10^{4}	1.373×10^{2}	1.438×10^{2}	–53.592	37.570	167.551	1.6650
134	135.448	5844.842	1017.530	7.938×10^{2}	3.637×10^{4}	1.539×10^{2}	1.616×10^{2}	–61.304	43.878	190.104	1.9683
135	100.446	7523.960	1018.019	1.728×10^{3}	8.523×10^{5}	1.532×10^{2}	1.596×10^{2}	–53.019	36.295	169.873	1.6257
136	150.475	6341.075	1112.183	8.569×10^{2}	3.861×10^{4}	1.684×10^{2}	1.772×10^{2}	–67.901	47.560	208.912	2.2523
137	103.035	5058.970	842.358	7.172×10^{2}	3.779×10^{4}	1.270×10^{2}	1.330×10^{2}	–45.804	31.441	152.780	1.5439
138	103.050	5059.032	842.405	7.173×10^{2}	3.780×10^{4}	1.270×10^{2}	1.331×10^{2}	–45.737	30.401	152.670	1.5483
139	114.754	5760.613	951.072	8.300×10^{2}	4.589×10^{4}	1.434×10^{2}	1.502×10^{2}	–50.049	33.257	172.969	1.7446
140	114.668	5759.885	951.005	8.298×10^{2}	4.588×10^{4}	1.434×10^{2}	1.502×10^{2}	–49.769	34.504	172.987	1.7446
141	114.702	5760.046	951.101	8.302×10^{2}	4.591×10^{4}	1.434×10^{2}	1.503×10^{2}	–49.649	31.149	172.746	1.7544
142	114.702	5760.052	951.100	8.298×10^{2}	4.589×10^{4}	1.434×10^{2}	1.503×10^{2}	–49.578	32.349	172.553	1.7541
143	109.471	5754.916	938.052	8.301×10^{2}	4.593×10^{4}	1.412×10^{2}	1.475×10^{2}	–47.986	32.745	166.985	1.6393
144	109.492	5755.007	938.103	8.302×10^{2}	4.594×10^{4}	1.412×10^{2}	1.475×10^{2}	–47.858	32.210	166.791	1.6372
145	127.550	7151.778	1142.398	1.056×10^{3}	6.218×10^{4}	1.718×10^{2}	1.792×10^{2}	–54.168	35.660	201.715	1.9341
146	127.548	7151.738	1142.394	1.056×10^{3}	6.218×10^{4}	1.718×10^{2}	1.792×10^{2}	–54.164	35.273	201.575	1.9350
147	127.581	7151.947	1142.496	1.056×10^{3}	6.216×10^{4}	1.717×10^{2}	1.792×10^{2}	–53.860	36.306	200.916	1.9313
148	127.581	7151.949	1142.497	1.056×10^{3}	6.217×10^{4}	1.717×10^{2}	1.792×10^{2}	–53.866	36.160	200.936	1.9314
149	143.593	6989.794	1168.156	9.878×10^{2}	5.148×10^{4}	1.762×10^{2}	1.845×10^{2}	–63.798	43.490	214.052	2.1352
150	96.994	4697.119	787.915	6.569×10^{2}	3.267×10^{4}	1.188×10^{2}	1.242×10^{2}	–40.445	25.981	143.772	1.4193
151	97.192	4698.126	788.075	6.568×10^{2}	3.266×10^{4}	1.188×10^{2}	1.243×10^{2}	–40.537	26.551	143.000	1.4433
152	82.237	6878.842	896.246	1.654×10^{3}	8.508×10^{5}	1.347×10^{2}	1.400×10^{2}	–39.986	26.755	146.080	1.3795
153	82.237	6878.834	896.244	1.654×10^{3}	8.508×10^{5}	1.347×10^{2}	1.400×10^{2}	–40.004	25.362	146.219	1.3791
154	107.086	4948.196	841.816	6.883×10^{2}	3.378×10^{4}	1.270×10^{2}	1.331×10^{2}	–44.267	29.578	155.092	1.5652
155	108.635	5398.064	896.565	7.694×10^{2}	4.076×10^{4}	1.351×10^{2}	1.414×10^{2}	–44.412	29.300	163.772	1.6202
156	112.775	5287.728	896.034	7.402×10^{2}	3.672×10^{4}	1.352×10^{2}	1.415×10^{2}	–43.298	29.176	166.538	1.6458
157	91.574	7968.738	1060.249	1.714×10^{3}	6.110×10^{5}	1.599×10^{2}	1.668×10^{2}	–36.798	22.586	171.250	2.4759
158	67.531	9060.687	1004.668	2.651×10^{3}	1.669×10^{6}	1.506×10^{2}	1.558×10^{2}	–39.501	26.002	148.494	1.3466
159	–54.479	23955.149	1242.020	2.183×10^{4}	1.467×10^{8}	1.866×10^{2}	1.918×10^{2}	–39.626	25.735	148.081	1.3343
160	6.612	16508.240	1123.384	1.224×10^{4}	7.418×10^{7}	1.686×10^{2}	1.738×10^{2}	–39.583	24.306	147.940	1.3390

Table S6. ***continued***

entry	S_S	I_F	S_{GBP}	E_2	E_3	R_2^r	R_3^r	G_1	G_2	G_3	I_G
161	6.581	16508.184	1123.362	1.224×10^4	7.418×10^7	1.686×10^2	1.738×10^2	–39.622	24.968	148.086	1.3468
162	92.314	7129.853	950.133	1.685×10^3	8.519×10^5	1.430×10^2	1.488×10^2	–43.791	30.205	157.630	1.5267
163	15.858	17597.808	1287.296	1.230×10^4	7.394×10^7	1.938×10^2	2.006×10^2	–36.483	22.012	173.366	2.4428
164	185.016	22439.487	2689.058	5.614×10^3	2.621×10^6	4.030×10^2	4.167×10^2	–81.050	44.993	428.582	3.6519
165	102.820	5038.009	842.241	7.090×10^2	3.564×10^4	1.269×10^2	1.327×10^2	–39.594	25.203	154.165	1.5187
166	102.578	5036.113	842.118	7.087×10^2	3.561×10^4	1.269×10^2	1.325×10^2	–38.898	25.173	154.779	1.4964
167	102.600	5036.203	842.121	7.087×10^2	3.561×10^4	1.269×10^2	1.325×10^2	–38.997	25.604	154.749	1.4946
168	102.984	5038.276	842.439	7.093×10^2	3.568×10^4	1.270×10^2	1.328×10^2	–39.349	13.866	153.420	1.5451
169	113.780	5714.458	950.536	8.130×10^2	4.154×10^4	1.431×10^2	1.492×10^2	–36.152	21.700	177.017	1.6474
170	107.201	4948.773	841.833	6.883×10^2	3.377×10^4	1.271×10^2	1.332×10^2	–44.816	29.465	155.530	1.5695
171	134.639	6241.280	1058.736	8.847×10^2	4.594×10^4	1.599×10^2	1.677×10^2	–51.560	33.928	197.753	2.0367
172	122.969	5539.346	949.948	7.719×10^2	3.783×10^4	1.435×10^2	1.504×10^2	–47.693	31.806	178.367	1.7955
173	163.756	6545.951	1165.776	8.985×10^2	4.229×10^4	1.767×10^2	1.860×10^2	–63.870	43.478	223.514	2.4087
174	95.675	5595.856	884.340	8.194×10^2	4.675×10^4	1.328×10^2	1.383×10^2	–41.081	26.603	154.759	1.4154
175	105.753	5846.870	938.239	8.508×10^2	4.786×10^4	1.411×10^2	1.471×10^2	–44.872	30.049	166.070	1.5645
176	106.827	6273.773	992.743	9.237×10^2	5.268×10^4	1.490×10^2	1.550×10^2	–38.032	22.773	176.922	1.5749
177	92.486	4805.599	788.272	6.855×10^2	3.668×10^4	1.187×10^2	1.240×10^2	–40.894	27.664	141.779	1.3790
178	92.752	4806.919	788.480	6.857×10^2	3.668×10^4	1.187×10^2	1.240×10^2	–40.947	27.603	140.629	1.3907
179	104.107	104.107	896.920	7.984×10^2	4.478×10^4	1.350×10^2	1.411×10^2	–44.760	29.973	161.705	1.5640
180	110.830	6203.857	992.796	9.112×10^2	5.292×10^4	1.492×10^2	1.557×10^2	–47.212	31.723	175.078	1.6828
181	98.058	5144.533	842.471	7.377×10^2	3.963×10^4	1.268×10^2	1.324×10^2	–39.401	25.621	152.937	1.4587
182	147.302	6877.336	1167.430	9.588×10^2	4.746×10^4	1.761×10^2	1.844×10^2	–61.682	41.749	216.746	2.1259
183	22.325	14773.508	1069.528	1.132×10^4	7.337×10^7	1.607×10^2	1.661×10^2	–39.017	23.551	155.223	1.4078
184	93.753	5253.977	842.981	7.669×10^2	4.368×10^4	1.267×10^2	1.321×10^2	–39.881	25.184	149.885	1.4127
185	93.553	5253.036	842.831	7.666×10^2	4.366×10^4	1.267×10^2	1.321×10^2	–39.756	26.231	150.786	1.4025
186	93.570	5253.092	842.832	7.666×10^2	4.365×10^4	1.267×10^2	1.321×10^2	–39.827	26.426	150.809	1.3987
187	93.598	5253.305	842.853	7.667×10^2	4.366×10^4	1.267×10^2	1.321×10^2	–39.885	25.977	150.633	1.4017
188	94.101	5255.780	843.250	7.671×10^2	4.370×10^4	1.268×10^2	1.322×10^2	–40.084	23.353	148.696	1.4391
189	94.103	5255.790	843.252	7.667×10^2	4.367×10^4	1.268×10^2	1.322×10^2	–40.021	25.652	148.645	1.4389
190	88.430	8525.818	1115.379	1.824×10^3	6.221×10^5	1.679×10^2	1.747×10^2	–36.301	19.462	177.535	2.4709
191	105.172	5953.872	951.468	8.794×10^2	5.175×10^4	1.431×10^2	1.492×10^2	–43.716	28.739	171.006	1.5949
192	223.941	11989.710	1940.801	1.798×10^3	1.101×10^5	2.930×10^2	3.068×10^2	–86.451	53.321	359.988	3.6584
193	216.966	11290.803	1844.860	1.685×10^3	1.020×10^5	2.787×10^2	2.918×10^2	–82.741	52.172	345.938	3.4781
194	88.275	8525.385	1115.287	1.824×10^3	6.220×10^5	1.679×10^2	1.748×10^2	–36.231	21.249	177.735	2.4798
195	97.724	8781.207	1168.937	1.856×10^3	6.233×10^5	1.754×10^2	1.822×10^2	–45.101	27.891	193.095	1.7751
196	103.758	5504.673	896.820	7.982×10^2	4.477×10^4	1.350×10^2	1.410×10^2	–43.736	29.225	161.832	1.5556
197	113.656	5755.001	950.588	8.298×10^2	4.588×10^4	1.433×10^2	1.499×10^2	–47.494	31.905	173.871	1.7046
198	104.750	5931.298	951.254	8.711×10^2	4.959×10^4	1.430×10^2	1.488×10^2	–36.933	21.884	172.897	1.5593
199	95.057	5702.647	897.713	8.478×10^2	5.064×10^4	1.348×10^2	1.403×10^2	–38.995	24.960	158.142	1.4670
200	94.812	5701.339	897.526	8.478×10^2	5.064×10^4	1.348×10^2	1.402×10^2	–38.819	24.580	159.113	1.4439
201	94.666	5700.710	897.410	8.478×10^2	5.064×10^4	1.348×10^2	1.402×10^2	–38.756	24.770	159.653	1.4237
202	94.904	5701.888	897.601	8.479×10^2	5.066×10^4	1.348×10^2	1.403×10^2	–38.848	24.169	158.661	1.4492

Table S6. *continued*

entry	S_S	I_F	S_{GBP}	E_2	E_3	R_2^r	R_3^r	G_1	G_2	G_3	I_G
203	105.101	5953.494	951.578	8.796×10^2	5.177×10^4	1.431×10^2	1.491×10^2	–42.870	26.745	169.958	1.6042
204	95.933	6149.099	952.120	9.289×10^2	5.762×10^4	1.428×10^2	1.482×10^2	–37.723	23.780	167.565	1.4525
205	95.765	6148.345	951.990	9.288×10^2	5.762×10^4	1.428×10^2	1.482×10^2	–37.652	23.452	168.436	1.4487
206	105.867	6399.488	1005.912	9.609×10^2	5.877×10^4	1.511×10^2	1.571×10^2	–41.583	23.448	180.129	1.5997
207	97.026	6596.679	1006.687	1.010×10^3	6.461×10^4	1.508×10^2	1.563×10^2	–36.701	21.867	176.660	1.4748
208	98.698	7047.134	1061.706	1.091×10^3	7.161×10^4	1.590×10^2	1.647×10^2	–36.018	20.110	183.643	1.5803
211	116.542	5791.156	964.431	8.119×10^2	4.116×10^4	1.453×10^2	1.520×10^2	–52.727	34.922	175.664	1.7159
212	141.757	5947.150	1043.787	8.146×10^2	3.823×10^4	1.581×10^2	1.664×10^2	–60.400	41.706	199.523	2.1105
213	141.817	5947.443	1043.894	8.146×10^2	3.822×10^4	1.581×10^2	1.664×10^2	–60.182	41.828	198.879	2.1287
214	113.219	5311.459	896.456	7.489×10^2	3.890×10^4	1.354×10^2	1.421×10^2	–49.428	34.129	163.029	1.7359
215	95.664	7886.685	1059.909	1.694×10^3	6.094×10^5	1.594×10^2	1.660×10^2	–47.096	29.920	174.317	1.7273
216	99.935	11415.322	1386.111	2.783×10^3	1.189×10^6	2.081×10^2	2.161×10^2	–52.336	33.468	216.031	2.1092
217	99.940	11415.336	1386.113	2.783×10^3	1.189×10^6	2.081×10^2	2.162×10^2	–52.365	31.967	216.236	2.1113
218	89.834	7525.846	1005.539	1.633×10^3	6.042×10^5	1.511×10^2	1.573×10^2	–41.981	27.245	165.025	1.6288
219	88.271	10693.602	1277.369	2.662×10^3	1.179×10^6	1.917×10^2	1.988×10^2	–42.169	25.152	197.378	1.9119
220	88.268	10693.579	1277.367	2.662×10^3	1.179×10^6	1.917×10^2	1.988×10^2	–42.194	23.827	197.493	1.9119
221	109.503	5755.892	938.057	8.304×10^2	4.598×10^4	1.412×10^2	1.477×10^2	–48.448	26.618	166.915	1.6574
222	86.151	7630.364	1006.139	1.662×10^3	6.081×10^5	1.518×10^2	1.585×10^2	–38.447	24.817	159.783	2.4086
223	86.151	7630.522	1006.210	1.662×10^3	6.081×10^5	1.518×10^2	1.586×10^2	–38.221	24.404	159.373	2.4339
224	80.937	10902.691	1278.556	2.720×10^3	1.187×10^6	1.930×10^2	2.013×10^2	–34.866	17.037	186.872	3.5116
225	80.919	10902.983	1278.699	2.720×10^3	1.186×10^6	1.931×10^2	2.015×10^2	–34.427	19.124	186.212	3.5621
226	80.924	10902.994	1278.700	2.719×10^3	1.186×10^6	1.931×10^2	2.015×10^2	–34.459	18.078	186.084	3.5604

Table S7. Eleven information-theoretic (ITA) descriptors for 1,4-naphthoquinone derivatives, calculated at the B3LYP/def2-TZVP level. All values are reported in atomic units.

entry	S_S	I_F	S_{GBP}	E_2	E_3	R_2^r	R_3^r	G_1	G_2	G_3	I_G
117	73.839	3958.830	640.353	5.622×10^{2}	2.937×10^{4}	9.610×10^{1}	1.003×10^{2}	–32.065	22.091	108.710	1.078
118	93.952	4460.721	748.122	6.255×10^{2}	3.160×10^{4}	1.127×10^{2}	1.180×10^{2}	–39.700	27.424	131.729	1.377
119	52.950	5555.966	666.143	1.475×10^{3}	8.433×10^{5}	9.990×10^{1}	1.037×10^{2}	–29.861	20.711	99.986	0.972
120	89.174	6650.288	882.227	1.622×10^{3}	8.496×10^{5}	1.330×10^{2}	1.389×10^{2}	–41.083	27.754	145.613	1.540
121	38.139	7737.472	774.457	2.472×10^{3}	1.661×10^{6}	1.158×10^{2}	1.194×10^{2}	–29.405	19.987	102.593	0.924
122	38.222	7737.755	774.521	2.472×10^{3}	1.661×10^{6}	1.158×10^{2}	1.195×10^{2}	–29.368	19.390	102.345	0.940
123	–83.827	22632.260	1011.909	2.165×10^{4}	1.467×10^{8}	1.518×10^{2}	1.554×10^{2}	–29.404	20.291	101.722	0.920
124	–73.617	22883.641	1065.907	2.168×10^{4}	1.467×10^{8}	1.601×10^{2}	1.644×10^{2}	–33.199	22.903	113.214	1.089
125	–63.612	23134.333	1119.748	2.171×10^{4}	1.467×10^{8}	1.684×10^{2}	1.732×10^{2}	–36.964	26.021	124.610	1.235
126	77.795	3625.321	611.695	5.098×10^{2}	2.633×10^{4}	9.227×10^{1}	9.681×10^{1}	–34.116	24.086	109.037	1.166
127	77.752	3625.327	611.643	5.099×10^{2}	2.635×10^{4}	9.227×10^{1}	9.680×10^{1}	–34.246	22.506	109.204	1.164
128	77.850	3625.590	611.741	5.098×10^{2}	2.633×10^{4}	9.228×10^{1}	9.683×10^{1}	–34.121	24.188	108.805	1.170
129	108.181	4379.657	773.364	6.049×10^{2}	2.968×10^{4}	1.172×10^{2}	1.235×10^{2}	–46.454	32.043	143.893	1.619
130	122.284	4874.543	867.546	6.678×10^{2}	3.189×10^{4}	1.316×10^{2}	1.389×10^{2}	–53.187	37.961	164.121	1.875
131	87.797	3876.009	665.536	5.414×10^{2}	2.744×10^{4}	1.005×10^{2}	1.056×10^{2}	–37.892	26.716	120.808	1.311
132	87.957	3876.705	665.666	5.415×10^{2}	2.744×10^{4}	1.006×10^{2}	1.057×10^{2}	–37.960	26.797	120.226	1.318
133	87.958	3876.713	665.667	5.414×10^{2}	2.744×10^{4}	1.006×10^{2}	1.057×10^{2}	–37.970	26.673	120.232	1.318
134	87.848	3876.419	665.561	5.414×10^{2}	2.744×10^{4}	1.006×10^{2}	1.056×10^{2}	–38.087	26.812	120.685	1.311
135	87.815	3876.453	665.509	5.414×10^{2}	2.744×10^{4}	1.005×10^{2}	1.056×10^{2}	–38.240	27.110	120.901	1.308
136	87.943	3876.730	665.648	5.414×10^{2}	2.744×10^{4}	1.006×10^{2}	1.057×10^{2}	–37.978	26.339	120.273	1.321
137	97.990	4127.998	719.499	5.731×10^{2}	2.855×10^{4}	1.089×10^{2}	1.146×10^{2}	–42.103	29.985	132.028	1.476
138	128.624	4883.288	881.455	6.680×10^{2}	3.190×10^{4}	1.338×10^{2}	1.413×10^{2}	–54.318	38.583	165.677	1.948
139	179.463	6141.730	1151.331	8.262×10^{2}	3.746×10^{4}	1.755×10^{2}	1.865×10^{2}	–73.716	51.357	223.710	2.825
140	189.664	6393.479	1205.314	8.578×10^{2}	3.858×10^{4}	1.838×10^{2}	1.954×10^{2}	–77.802	54.240	234.962	2.983
141	159.141	5877.470	1071.532	7.943×10^{2}	3.636×10^{4}	1.626×10^{2}	1.719×10^{2}	–67.373	48.429	201.536	2.375
142	443.230	13640.099	2666.685	1.775×10^{3}	7.094×10^{4}	4.073×10^{2}	4.338×10^{2}	–182.829	129.116	527.423	6.799
143	524.552	15892.745	3126.549	2.059×10^{3}	8.096×10^{4}	4.776×10^{2}	5.088×10^{2}	–215.782	154.782	618.646	7.992
144	150.284	10506.034	1368.186	2.821×10^{3}	1.674×10^{6}	2.072×10^{2}	2.175×10^{2}	–73.834	52.014	226.291	2.650
145	97.861	4127.232	719.407	5.730×10^{2}	2.855×10^{4}	1.088×10^{2}	1.145×10^{2}	–41.915	29.922	132.301	1.459
146	108.053	4378.550	773.416	6.047×10^{2}	2.967×10^{4}	1.171×10^{2}	1.234×10^{2}	–45.608	32.271	143.427	1.613
147	79.312	4074.970	666.426	5.911×10^{2}	3.334×10^{4}	1.004×10^{2}	1.051×10^{2}	–34.165	22.500	117.711	1.217
148	79.356	4075.468	666.446	5.910×10^{2}	3.332×10^{4}	1.004×10^{2}	1.051×10^{2}	–34.398	23.237	117.743	1.219
149	80.664	4523.850	721.200	6.721×10^{2}	4.031×10^{4}	1.084×10^{2}	1.132×10^{2}	–33.259	21.911	125.802	1.266
150	90.917	4776.109	775.066	7.037×10^{2}	4.142×10^{4}	1.168×10^{2}	1.223×10^{2}	–38.300	25.202	138.073	1.421
151	90.963	4776.081	775.081	7.037×10^{2}	4.142×10^{4}	1.168×10^{2}	1.223×10^{2}	–38.228	25.673	138.030	1.423
152	85.766	4771.086	762.117	7.039×10^{2}	4.147×10^{4}	1.145×10^{2}	1.196×10^{2}	–36.471	24.347	132.180	1.313
153	86.850	5218.580	816.682	7.850×10^{2}	4.845×10^{4}	1.226×10^{2}	1.277×10^{2}	–35.550	23.071	141.150	1.334
154	102.441	5719.238	911.760	8.484×10^{2}	5.072×10^{4}	1.371×10^{2}	1.431×10^{2}	–43.136	28.607	157.847	1.581
155	121.891	7564.636	1170.808	1.155×10^{3}	7.395×10^{4}	1.757×10^{2}	1.829×10^{2}	–48.764	30.892	201.140	1.901
156	94.702	4572.542	761.532	6.542×10^{2}	3.556×10^{4}	1.148×10^{2}	1.204×10^{2}	–40.820	28.185	135.501	1.433
157	99.712	4578.127	774.367	6.543×10^{2}	3.556×10^{4}	1.170×10^{2}	1.229×10^{2}	–42.130	28.665	140.209	1.524

Table S7. *continued*

entry	S_S	I_F	S_{GBP}	E_2	E_3	R_2^r	R_3^r	G_1	G_2	G_3	I_G
158	99.312	7682.241	1046.212	1.644×10^{3}	6.035×10^{5}	1.574×10^{2}	1.641×10^{2}	–49.524	33.671	172.368	1.730
159	73.354	3713.426	612.052	5.304×10^{2}	2.818×10^{4}	9.212×10^{1}	9.630×10^{1}	–28.749	19.085	108.452	1.105
160	63.646	5637.552	720.238	1.346×10^{3}	5.866×10^{5}	1.082×10^{2}	1.124×10^{2}	–28.758	15.842	113.943	1.119
161	95.478	4999.931	815.754	7.271×10^{2}	4.040×10^{4}	1.227×10^{2}	1.281×10^{2}	–34.015	21.419	146.020	1.418
162	105.660	5251.668	869.718	7.588×10^{2}	4.152×10^{4}	1.310×10^{2}	1.370×10^{2}	–38.141	24.201	157.307	1.575
163	99.668	4557.953	774.211	6.460×10^{2}	3.339×10^{4}	1.169×10^{2}	1.226×10^{2}	–36.929	23.632	142.691	1.500
164	93.768	4217.059	719.846	5.936×10^{2}	3.041×10^{4}	1.087×10^{2}	1.142×10^{2}	–37.776	25.819	132.096	1.407
165	95.759	5001.799	815.906	7.271×10^{2}	4.040×10^{4}	1.228×10^{2}	1.283×10^{2}	–34.822	21.899	145.679	1.455
166	68.170	5551.088	720.018	1.325×10^{3}	5.853×10^{5}	1.083×10^{2}	1.130×10^{2}	–34.540	23.121	113.906	1.195
167	78.259	5802.276	773.884	1.357×10^{3}	5.860×10^{5}	1.166×10^{2}	1.219×10^{2}	–38.534	26.970	125.409	1.351
168	88.481	6054.096	827.868	1.388×10^{3}	5.871×10^{5}	1.249×10^{2}	1.308×10^{2}	–42.608	29.814	136.634	1.510
169	68.898	3822.238	612.430	5.594×10^{2}	3.221×10^{4}	9.204×10^{1}	9.607×10^{1}	–29.444	19.441	106.415	1.053
170	68.783	3821.779	612.339	5.596×10^{2}	3.223×10^{4}	9.204×10^{1}	9.607×10^{1}	–29.418	18.383	106.843	1.050
171	78.971	4073.257	666.332	5.910×10^{2}	3.333×10^{4}	1.003×10^{2}	1.049×10^{2}	–33.274	22.165	117.822	1.201
172	89.167	4325.159	720.301	6.226×10^{2}	3.444×10^{4}	1.086×10^{2}	1.138×10^{2}	–37.302	25.465	129.040	1.353
173	119.805	5080.455	882.259	7.177×10^{2}	3.778×10^{4}	1.335×10^{2}	1.406×10^{2}	–49.539	33.807	162.596	1.826
174	130.019	5332.227	936.248	7.492×10^{2}	3.889×10^{4}	1.418×10^{2}	1.495×10^{2}	–53.599	37.101	173.909	1.983
175	140.233	5583.998	990.236	7.809×10^{2}	4.001×10^{4}	1.501×10^{2}	1.584×10^{2}	–57.663	39.989	185.126	2.141
176	150.449	5835.769	1044.227	8.125×10^{2}	4.112×10^{4}	1.585×10^{2}	1.673×10^{2}	–61.697	43.258	196.357	2.298
177	160.663	6087.539	1098.214	8.441×10^{2}	4.223×10^{4}	1.668×10^{2}	1.762×10^{2}	–65.752	46.278	207.553	2.456
178	181.088	6591.078	1206.185	9.073×10^{2}	4.445×10^{4}	1.834×10^{2}	1.941×10^{2}	–73.858	52.521	229.911	2.770
179	119.747	5080.103	882.189	7.175×10^{2}	3.777×10^{4}	1.335×10^{2}	1.405×10^{2}	–49.473	34.413	162.876	1.818
180	114.537	5073.512	869.249	7.176×10^{2}	3.781×10^{4}	1.313×10^{2}	1.379×10^{2}	–47.780	32.156	158.252	1.714
181	170.701	6338.483	1152.006	8.757×10^{2}	4.335×10^{4}	1.750×10^{2}	1.851×10^{2}	–69.778	49.033	219.446	2.600
182	189.237	8241.026	1425.100	1.146×10^{3}	5.705×10^{4}	2.155×10^{2}	2.264×10^{2}	–81.593	57.268	265.068	2.812
183	189.340	8241.401	1425.208	1.146×10^{3}	5.706×10^{4}	2.155×10^{2}	2.264×10^{2}	–81.528	56.480	264.690	2.811
184	193.296	7559.931	1355.277	1.033×10^{3}	4.893×10^{4}	2.057×10^{2}	2.170×10^{2}	–82.742	59.002	258.547	2.927
185	95.304	15969.268	1379.329	1.146×10^{4}	7.337×10^{7}	2.091×10^{2}	2.192×10^{2}	–69.588	49.195	220.503	2.626
186	178.160	6823.027	1231.984	9.385×10^{2}	4.557×10^{4}	1.873×10^{2}	1.980×10^{2}	–75.398	53.264	239.550	2.750
187	108.504	7480.954	1045.756	1.596×10^{3}	5.986×10^{5}	1.574×10^{2}	1.643×10^{2}	–51.862	34.777	174.727	1.764
188	263.226	10782.782	1800.347	2.007×10^{3}	6.130×10^{5}	2.746×10^{2}	2.917×10^{2}	–109.696	75.306	338.434	4.418
189	210.494	9998.754	1585.575	1.912×10^{3}	6.096×10^{5}	2.409×10^{2}	2.547×10^{2}	–91.274	62.172	289.822	3.538
190	265.919	9770.863	1787.821	1.336×10^{3}	6.373×10^{4}	2.723×10^{2}	2.890×10^{2}	–109.609	71.953	350.806	4.276
191	115.276	5984.755	979.224	8.536×10^{2}	4.494×10^{4}	1.473×10^{2}	1.538×10^{2}	–47.253	30.818	174.780	1.677
192	246.534	8918.014	1639.444	1.212×10^{3}	5.637×10^{4}	2.492×10^{2}	2.637×10^{2}	–100.376	70.737	316.052	3.733
193	287.344	9924.823	1855.371	1.339×10^{3}	6.082×10^{4}	2.825×10^{2}	2.994×10^{2}	–116.625	82.221	361.120	4.366
194	80.549	4523.249	721.129	6.721×10^{2}	4.031×10^{4}	1.084×10^{2}	1.132×10^{2}	–33.293	21.719	126.254	1.255
195	165.805	6778.996	1192.878	9.567×10^{2}	5.034×10^{4}	1.810×10^{2}	1.909×10^{2}	–68.344	46.778	226.425	2.578
196	–6.917	13450.968	839.454	1.115×10^{4}	7.336×10^{7}	1.260×10^{2}	1.298×10^{2}	–28.982	18.524	108.901	1.005
197	78.885	4072.873	666.264	5.909×10^{2}	3.331×10^{4}	1.003×10^{2}	1.049×10^{2}	–33.213	22.429	118.277	1.200
198	78.884	4072.902	666.262	5.909×10^{2}	3.332×10^{4}	1.003×10^{2}	1.050×10^{2}	–33.221	22.229	118.363	1.203
199	70.212	4271.014	667.165	6.405×10^{2}	3.920×10^{4}	1.001×10^{2}	1.042×10^{2}	–28.428	18.196	114.428	1.095

Table S7. *continued*

entry	S_S	I_F	S_{GBP}	E_2	E_3	R_2^r	R_3^r	G_1	G_2	G_3	I_G
200	69.849	4269.156	666.900	6.405×10^2	3.919×10^4	1.000×10^2	1.041×10^2	–28.220	18.094	115.471	1.061
201	182.438	7039.953	1260.958	9.884×10^2	5.145×10^4	1.914×10^2	2.022×10^2	–73.079	50.832	238.073	2.823
202	93.907	6117.295	938.862	9.472×10^2	6.238×10^4	1.408×10^2	1.463×10^2	–33.402	19.405	163.387	1.501
203	107.307	7032.993	991.113	1.514×10^3	5.916×10^5	1.494×10^2	1.562×10^2	–52.778	37.536	165.981	1.736
204	131.044	8016.788	1167.019	1.641×10^3	5.960×10^5	1.760×10^2	1.842×10^2	–64.398	46.116	201.178	2.065
205	62.445	6646.613	830.256	1.536×10^3	6.036×10^5	1.252×10^2	1.306×10^2	–26.798	15.430	124.611	2.094
206	68.810	3824.071	612.127	5.596×10^2	3.226×10^4	9.206×10^1	9.613×10^1	–30.683	19.631	106.713	1.062
207	97.026	6596.679	1006.687	1.010×10^3	6.461×10^4	1.508×10^2	1.563×10^2	–36.701	21.867	176.660	1.4748
208	98.698	7047.134	1061.706	1.091×10^3	7.161×10^4	1.590×10^2	1.647×10^2	–36.018	20.110	183.643	1.5803
211	116.542	5791.156	964.431	8.119×10^2	4.116×10^4	1.453×10^2	1.520×10^2	–52.727	34.922	175.664	1.7159
212	141.757	5947.150	1043.787	8.146×10^2	3.823×10^4	1.581×10^2	1.664×10^2	–60.400	41.706	199.523	2.1105
213	141.817	5947.443	1043.894	8.146×10^2	3.822×10^4	1.581×10^2	1.664×10^2	–60.182	41.828	198.879	2.1287
214	113.219	5311.459	896.456	7.489×10^2	3.890×10^4	1.354×10^2	1.421×10^2	–49.428	34.129	163.029	1.7359
215	95.664	7886.685	1059.909	1.694×10^3	6.094×10^5	1.594×10^2	1.660×10^2	–47.096	29.920	174.317	1.7273
216	99.935	11415.322	1386.111	2.783×10^3	1.189×10^6	2.081×10^2	2.161×10^2	–52.336	33.468	216.031	2.1092
217	99.940	11415.336	1386.113	2.783×10^3	1.189×10^6	2.081×10^2	2.162×10^2	–52.365	31.967	216.236	2.1113
218	89.834	7525.846	1005.539	1.633×10^3	6.042×10^5	1.511×10^2	1.573×10^2	–41.981	27.245	165.025	1.6288
219	88.271	10693.602	1277.369	2.662×10^3	1.179×10^6	1.917×10^2	1.988×10^2	–42.169	25.152	197.378	1.9119
220	88.268	10693.579	1277.367	2.662×10^3	1.179×10^6	1.917×10^2	1.988×10^2	–42.194	23.827	197.493	1.9119
221	109.503	5755.892	938.057	8.304×10^2	4.598×10^4	1.412×10^2	1.477×10^2	–48.448	26.618	166.915	1.6574
222	86.151	7630.364	1006.139	1.662×10^3	6.081×10^5	1.518×10^2	1.585×10^2	–38.447	24.817	159.783	2.4086
223	86.151	7630.522	1006.210	1.662×10^3	6.081×10^5	1.518×10^2	1.586×10^2	–38.221	24.404	159.373	2.4339
224	80.937	10902.691	1278.556	2.720×10^3	1.187×10^6	1.930×10^2	2.013×10^2	–34.866	17.037	186.872	3.5116
225	80.919	10902.983	1278.699	2.720×10^3	1.186×10^6	1.931×10^2	2.015×10^2	–34.427	19.124	186.212	3.5621
226	80.924	10902.994	1278.700	2.719×10^3	1.186×10^6	1.931×10^2	2.015×10^2	–34.459	18.078	186.084	3.5604

Table S8. Eleven information-theoretic (ITA) descriptors for miscellaneous quinone and nonquinone derivatives, calculated at the B3LYP/def2-TZVP level. All values are reported in atomic units.

entry	S_S	I_F	S_{GBP}	E_2	E_3	R_2^r	R_3^r	G_1	G_2	G_3	I_G
317	–15.096	11116.941	815.230	4.340×10^3	3.293×10^6	1.210×10^2	1.229×10^2	–17.054	11.517	72.579	0.491
318	–259.075	40906.474	1290.103	4.270×10^4	2.933×10^8	1.930×10^2	1.949×10^2	–17.022	11.873	70.871	0.491
319	125.458	4403.306	813.588	6.052×10^2	2.967×10^4	1.237×10^2	1.312×10^2	–50.915	35.866	152.754	1.933
320	97.704	4699.236	788.345	6.567×10^2	3.267×10^4	1.188×10^2	1.243×10^2	–40.786	27.900	142.036	1.427
321	–7.890	13003.761	784.955	1.106×10^4	7.335×10^7	1.179×10^2	1.217×10^2	–29.912	20.839	99.210	0.968
322	94.727	4572.675	761.547	6.542×10^2	3.557×10^4	1.148×10^2	1.204×10^2	–40.863	27.987	135.435	1.433
323	93.910	4217.892	719.909	5.937×10^2	3.042×10^4	1.087×10^2	1.142×10^2	–38.018	25.706	131.660	1.414
324	93.886	4217.463	720.014	5.938×10^2	3.042×10^4	1.087×10^2	1.142×10^2	–37.387	25.081	131.066	1.413
325	69.058	3823.003	612.536	5.594×10^2	3.221×10^4	9.204×10^1	9.604×10^1	–29.429	19.892	105.632	1.058
326	62.367	6646.373	830.190	1.536×10^3	6.036×10^5	1.252×10^2	1.306×10^2	–26.747	16.245	124.824	2.092
327	57.186	9918.909	1102.710	2.594×10^3	1.182×10^6	1.665×10^2	1.735×10^2	–22.993	9.919	151.364	3.222
328	118.628	5557.149	937.612	7.803×10^2	4.003×10^4	1.414×10^2	1.484×10^2	–52.442	36.895	169.817	1.764
329	92.796	4806.707	788.494	6.858×10^2	3.669×10^4	1.187×10^2	1.241×10^2	–40.999	26.786	140.931	1.384
330	132.076	5364.329	949.493	7.309×10^2	3.414×10^4	1.438×10^2	1.514×10^2	–57.910	41.627	177.766	1.950
331	94.169	5256.107	843.300	7.667×10^2	4.367×10^4	1.268×10^2	1.322×10^2	–40.219	26.444	148.526	1.446
332	123.357	4876.262	868.499	6.678×10^2	3.191×10^4	1.316×10^2	1.387×10^2	–52.375	35.451	161.139	1.839
333	204.347	6888.185	1299.580	9.208×10^2	4.081×10^4	1.981×10^2	2.102×10^2	–84.964	61.600	253.161	3.110
334	245.551	7897.168	1515.941	1.047×10^3	4.527×10^4	2.313×10^2	2.459×10^2	–101.520	73.417	296.825	3.760
335	86.423	4110.075	692.845	5.726×10^2	2.853×10^4	1.045×10^2	1.094×10^2	–38.621	27.107	126.676	1.267
336	88.824	5006.523	802.163	7.349×10^2	4.250×10^4	1.206×10^2	1.257×10^2	–36.714	24.004	143.786	1.346
337	75.241	5586.552	746.346	1.307×10^3	5.803×10^5	1.125×10^2	1.174×10^2	–39.367	26.968	123.333	1.261
338	81.988	6044.861	814.141	1.388×10^3	5.869×10^5	1.228×10^2	1.284×10^2	–41.173	28.722	135.042	1.453
339	77.497	6487.688	855.504	1.468×10^3	5.931×10^5	1.288×10^2	1.342×10^2	–39.929	27.183	140.362	1.421
340	97.263	4807.804	801.134	6.854×10^2	3.662×10^4	1.208×10^2	1.263×10^2	–40.909	28.140	147.234	1.433
341	115.294	4415.486	799.721	5.864×10^2	2.488×10^4	1.213×10^2	1.280×10^2	–51.275	36.676	152.757	1.700
342	110.075	7688.033	1072.235	1.644×10^3	6.033×10^5	1.625×10^2	1.707×10^2	–47.638	32.070	179.457	2.783
343	85.143	3661.883	638.243	4.916×10^2	2.155×10^4	9.641×10^1	1.012×10^2	–39.035	27.529	117.179	1.231
344	111.129	4171.733	759.181	5.549×10^2	2.377×10^4	1.152×10^2	1.217×10^2	–48.773	35.349	144.560	1.666
345	101.595	4607.219	787.821	6.358×10^2	3.076×10^4	1.189×10^2	1.247×10^2	–45.200	31.928	144.691	1.487
346	97.889	4367.984	747.645	6.047×10^2	2.969×10^4	1.128×10^2	1.185×10^2	–43.543	30.639	134.181	1.442
347	85.506	4327.697	710.479	5.887×10^2	2.554×10^4	1.062×10^2	1.106×10^2	–33.488	23.225	115.480	1.109
348	79.946	4724.473	748.296	6.872×10^2	3.716×10^4	1.123×10^2	1.167×10^2	–25.531	14.509	133.556	1.186
349	85.640	3645.029	638.202	4.834×10^2	1.941×10^4	9.639×10^1	1.012×10^2	–35.685	25.011	119.635	1.219

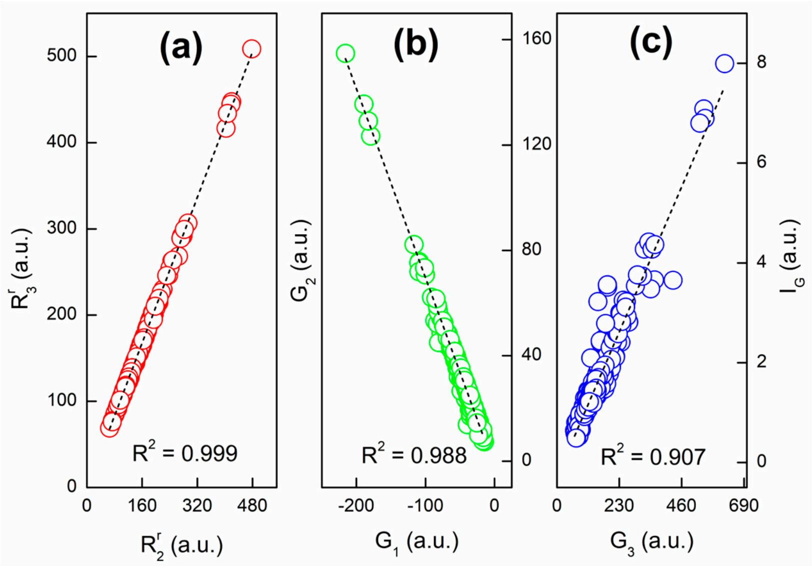

Figure S1. Intercorrelations between ITA quantities: (a) R^r_3 vs R^r_2, (b) G_2 vs G_1, and (c) I_G vs G_3 for all the species considered in this work.
